# Research status and challenges of anti-tumor effects of natural saponins and their combination therapy

**DOI:** 10.1186/s43046-026-00354-x

**Published:** 2026-05-18

**Authors:** Can Cui, Zhou-Juan Zheng, Si Li, Xiang-Lan Piao

**Affiliations:** School of Pharmacy, Minzu University of China, Beijing, China

**Keywords:** Traditional medicine, Saponins, Anti-tumor, Chemotherapy drugs, Combination drugs

## Abstract

Cancer is a leading global cause of death, and traditional chemotherapy is plagued by severe side effects, making the development of high-efficacy and low-toxicity antitumor agents an urgent need. Saponins, natural bioactive compounds, exhibit prominent antitumor potential. This review systematically retrieved relevant literature from 2015 to 2025 via Web of Science, PubMed and other databases, summarizing the antitumor effects and mechanisms of saponins, their synergistic value in combination with chemotherapy, as well as existing saponin-based drugs, translational challenges and corresponding solutions. Saponin monomers exert distinct antitumor efficacy, and their rational combination with chemotherapy yields notable advantages. Structural modification and dosage form optimization can effectively mitigate the inherent limitations of saponins. In conclusion, natural saponins hold great translational potential for antitumor therapy, and combination regimens represent a promising research focus in oncology treatment.

**Graphical abstract:**

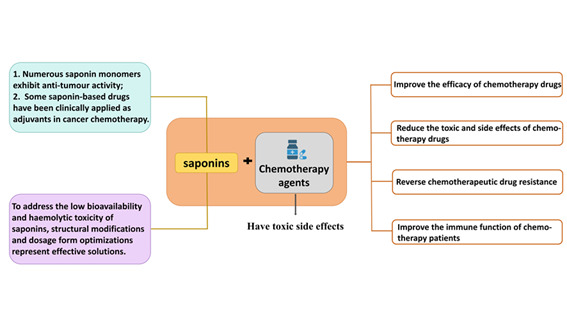

**Supplementary Information:**

The online version contains supplementary material available at 10.1186/s43046-026-00354-x.

## Highlight

- Summarizes the role of saponin monomers in the antitumor field over the past decade.
- Saponins enhance chemotherapy efficacy through four pathways, namely therapeutic effect improvement, toxicity reduction, drug resistance reversal, and immune enhancement.
- Summarizes the roles of saponin-based drugs currently available in the Chinese market in antitumor therapy and adjuvant chemotherapy.
- The low bioavailability and hemolytic toxicity of saponins can be effectively improved via structural modification and formulation optimization.

**Supplementary Information:**

The online version contains supplementary material available at 10.1186/s43046-026-00354-x.

## Introduction

**Fig. 1:**
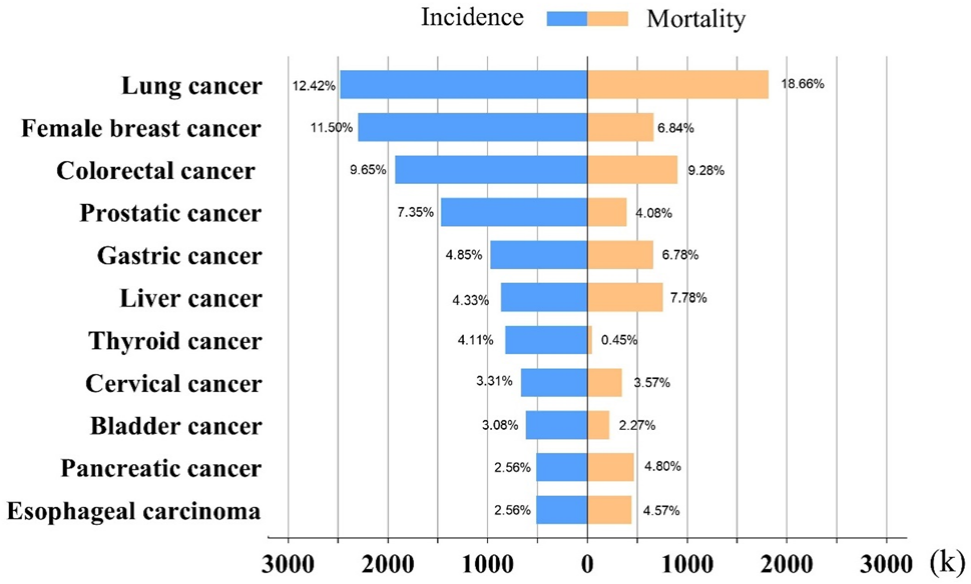
The incidence and mortality rates of different types of cancer globally. *The data sourced from the 2024 Global Cancer Statistics Report*

With ongoing global economic and socioeconomic development, multiple factors have led to a gradual increase in cancer incidence worldwide. The 2024 Global Cancer Statistics Report by the International Agency for Research on Cancer (IARC) [1] indicates that approximately 20 million new cancer cases were diagnosed globally in 2022, with 9.7 million cancer-related deaths; lung, breast, colorectal, and prostate cancers each exceeded 1 million new cases (Fig. 1). The National Cancer Center and the IARC published 2024 national cancer incidence and mortality statistics [2]. Data indicated that cancer incidence was low among individuals younger than 35 years, increased markedly in the 35–39 age group (125.24 per 100,000 population), and peaked at more than 80 years of age (1,461.63 per 100,000 population). Without breakthroughs in cancer prevention and treatment, global cancer incidence and mortality will continue to rise alongside extended life expectancy and population aging, severely affecting patients’ lives and imposing a substantial socioeconomic burden. A collaborative study involving Peking Union Medical College, Tsinghua University, and international experts [3] projected that the global economic cost of cancer from 2020 to 2050 will reach 25.2 trillion USD, accounting for 0.55% of annual global Gross Domestic Product (GDP); the United States and China bear the highest economic losses, accounting for 24.1% and 20.8% of the total global burden, respectively. Developing effective and low-toxicity cancer therapies thus remains an urgent public health priority. Current cancer treatment strategies have become increasingly refined, with emerging approaches such as targeted therapy and immunotherapy increasingly integrated into clinical practice. Nevertheless, each of these modalities presents specific limitations, including adverse effects, high costs, and restricted efficacy in certain patient populations [4, 5].

Currently, chemotherapy remains the most common clinical cancer treatment, but its toxic effects on normal cells impair patients’ physiological and immune functions, leading to severe complications [6]. Compounding these challenges, studies have shown that chemotherapy drugs, while effective in killing cancer cells, may also promote cancer cell metastasis [7]. Research indicates that chemotherapy drugs such as paclitaxel can remodel the tumor microenvironment and enhance the invasive and distant metastatic capabilities of breast cancer cells by increasing the density and activity of the metastatic tumor microenvironment (TME) sites and upregulating the expression of the actin regulatory protein MENA [8]. Furthermore, a study has shown that both paclitaxel and doxorubicin—chemotherapeutic agents with fundamentally different mechanisms of action—stimulate increased exosome production in cancer cells, elevate intracellular Ca²⁺ levels, and promote the translocation of annexin A6 into exosomes. These annexin A6-containing exosomes can reach the lungs via the circulatory system, induce the expression of the chemokine CCL2 in lung tissue, and thereby promote cancer cell lung metastasis [9]. These findings suggest that such pro-metastatic effects may be common during chemotherapy. Additionally, high-dose or long-term chemotherapy often induces drug resistance in cancer cells, diminishing therapeutic efficacy and posing a major challenge for chemotherapy. The complexity of resistance mechanisms [10] further hinders the optimization of treatment regimens. When drugs fail to effectively inhibit or kill cancer cells, the risk of cancer cell proliferation significantly increases [11]. This leads to tumor recurrence or metastasis and subsequently reduces patients’ cure rates and survival rates.

Saponins, an important class of active ingredients in natural medicines, consist of hydrophobic aglucones and hydrophilic sugar chains connected by glycosidic bonds. With a typical amphiphilic structure, they can be categorized into triterpenoid saponins and steroidal saponins according to their aglucones. Widely distributed in terrestrial higher plants and certain marine organisms [12], saponins have garnered extensive attention due to their diverse biological activities, including anti-inflammatory [13], antioxidant [14], lipid-lowering [15] effects, and other pharmacological properties, as well as their potential application value. Notably, numerous studies have demonstrated that various saponins exhibit distinct anti-tumor effects, such as ginsenosides [16], gypenosides [17], notoginsenosides [18], and astragalosides [19]. Validated through cell experiments, animal models, or clinical studies, these saponins show inhibitory effects against various tumors. Saponins can bind to cholesterol in cell membranes, forming channels that induce membrane permeabilization and disrupt the barrier function of the cell membrane. This triggers intracellular apoptosis/necrosis signaling pathways, ultimately exerting cytotoxicity against cancer cells [20–22]. However, some literature indicates that certain saponins exhibit significant toxicity toward cancer cells while causing relatively mild toxicity to normal cells. This may stem from the abnormal, heightened cholesterol metabolism in cancer cells—driven by the synthesis of cell membrane components and other functional demands [23]. This characteristic renders it highly sensitive to saponins, ultimately leading to the binding and inactivation of cholesterol synthesized within cancer cells, thereby inhibiting their growth [15, 24]. There are also research indicates that normal cells primarily obtain energy through aerobic metabolism by breaking down glucose, whereas cancer cells rely on anaerobic metabolism (i.e., glycolysis) to derive energy from glucose [25]. Certain saponins can inhibit glycolysis in cancer cells while promoting aerobic metabolism in normal cells, thereby suppressing cancer cell growth and proliferation and inducing apoptosis [26, 27]. Therefore, saponins targeting aberrant pathways such as cholesterol metabolism and glycolysis in cancer cells exhibit significant tumor-selective cytotoxicity.

Chemotherapy drugs and saponins can both play roles in cancer treatment. When used in rational combination, saponins can serve as an adjunct therapy by reducing the dosage of chemotherapy drugs, thereby lowering toxic side effects and enhancing efficacy [28]. The combined application of natural products such as saponins and chemotherapy drugs has also become a research hotspot in the fields of medicine and pharmacy in recent years.

This review systematically retrieved relevant literature published from 2015 to 2025 from databases including Web of Science and PubMed, using the keywords “saponins,” “tumors,” and “combination therapy.” Building upon this foundation, this review comprehensively summarizes the antitumor effects of saponins against various malignant tumors and their underlying mechanisms, while exploring their synergistic therapeutic potential in combination with chemotherapeutic agents. From a translational medicine perspective, this work uniquely synthesizes a decade of research findings, focusing not only on mechanisms of action but also on clinical applications, the theoretical basis of combination therapies, and key challenges limiting the clinical translation of saponins. It further proposes feasible strategies to address these obstacles. This review aims to provide a theoretical foundation and innovative directions for the further development and clinical application of natural saponins in antitumor therapy, particularly in combination chemotherapy.

## The anti-tumor effect of natural saponin monomers

Previous studies have demonstrated that various saponins indeed exert therapeutic effects in cancer treatment through multiple mechanisms [29, 30]. These articles predominantly categorise their findings based on anti-cancer mechanisms, whilst the classification of targeted cancer types remains unclear. Building on the promising antitumor potential of natural saponins and the urgent need for low-toxicity antitumor agents, this article systematically collected and organized relevant literature. The high-impact literatures focused on the anti-tumor effects of various saponin (limited to monomeric compounds) on different types of cancer over the past decade by comprehensively searching authoritative academic journal databases such as PubMed and Web of Science. We selected representative saponins and classified them in accordance with their chemical structural characteristics and natural sources. To facilitate the direct identification of potent saponins against specific cancer types and their underlying mechanisms of action, a concise summary table (Table 1) featuring saponins with a broad spectrum of anti-tumor activity across multiple cancer types, synergistic in vitro and in vivo anti-cancer efficacy as well as unique action mechanisms is presented in the main text. Meanwhile, a comprehensive compilation of the anti-tumor activities of triterpenoid saponins and steroidal saponins is provided in Supplementary Table S1 and S2 for detailed reference.

**Table 1:** Promising Antitumor Saponins: Multi-Cancer Efficacy

Class	Family name	Source	Component	Cancer species	Effect	References
Triterpenoid saponins	Araliaceae	*Panax ginseng*	Ginsenoside CK	Gastric cancer	Cytotoxic, inducing cancer cell apoptosis. Bax、Caspase-3 ↑ CyclinB1、CyclinD1、Bcl-2、p-PI3K、p-AKT、NF-κB p65、p-IκBα ↓	[31]
				Liver cancer	Cytotoxicity, inhibition of cancer cell proliferation, G0/G1 phase arrest, suppression of ATP production, inhibition of cellular glycolysis, and suppression of tumor growth in experimental animals. PHD、RACK1 ↑ Bclaf1、HIF-1α、HSP70、HSP90、pVHL、GLUT1、HK2、LDHA、PDK1 ↓	[32]
					Cytotoxicity, induction of cancer cell apoptosis, inhibition of glycolysis. pro-Caspase 3、pro-PARP、p-AKT、p-mTOR、c-Myc、HK2、PKM2 ↓	[33]
					Cytotoxicity, inhibition of cancer cell proliferation, increased thermal stability of Annexin A2, reduced interaction between Annexin A2 and p50. Caspase 3、Caspase 9 ↑ IL-6、X-IAP, c-IAP1, c-IAP2、Survivin ↓	[34]
				Lung cancer	Cytotoxicity, induction of cancer cell apoptosis, inhibition of tyrosine kinase activity, and suppression of tumor growth in experimental animals. Caspase-9 ↑ EGFR、p-EGFR、p-B-RAF、p-C-RAF、p-ERK、p-MEK、p-STAT3、BcL-Xl、Survivin ↓	[35]
			Ginsenoside Rg3	Colon cancer	Cytotoxicity, inhibition of cancer cell migration and invasion, suppression of EMT, and inhibition of tumor growth and metastasis in experimental animals. E-cadherin ↑ Vimentin、Snail、NICD、Hes1、Notch1、Notch2、Notch3 ↓	[36]
					Cytotoxicity, induce mitochondrial damage and autophagy in cancer cells, enhance the interaction between PINK1 and Parkin proteins. LC3-II、PINK1、Parkin、GAPDH、p62、ubiquitinated GAPDH ↑ TIM23 、VDAC1、MFN2、ULK1 ↓	[37]
				Melanoma	Cytotoxicity, inhibition of cancer cell proliferation, inhibition of tumor growth in experimental animals. FUT4、Lewis Y、UEA、p-EGFR、p-ERK1/2 ↓	[38]
				NSCLC	Cytotoxicity, minor DNA damage. VRK1、P53BP1 ↑	[39]
	Campanulaceae	*Platycodon grandiflorus*	Platycodin D	Acute myeloid leukemia	Cytotoxicity, induction of cancer cell apoptosis, reduction of mitochondrial membrane potential, G0/G1 cycle arrest. Cleaved-PARP、Cleaved-Caspase-3、cytosolic cytochrome C、BAK、BIM ↑ p-Akt、p-GSK3β、p-S6、p-ERK ↓	[40]
				Adrenal pheochromocytoma	Cytotoxicity, induction of cancer cell apoptosis, induction of DNA fragmentation, induction of mitochondrial membrane potential reduction, G0/G1 cycle arrest, induction of autophagy. ROS、Caspase 3、Bid ↑ pro-Caspase 7、Bcl-2、Bcl-xL ↓	[41]
				Liver cancer	Cytotoxicity, inducing escape of endocytosed EGFP-HBP from endosomes while damaging vesicle membranes, promoting redistribution of free cholesterol to enhance MHBP’s antitumor activity, and facilitating cancer cell apoptosis. MHBP、p-JNK、p-p38、Cleaved-Caspase-3、Cleaved-Caspase-8、Cleaved-Caspase-9 ↑ cathepsin B、cathepsin D、p-Akt、p-ERK1/2 ↓	[42]
					Cytotoxicity, inducing apoptosis and autophagy in cancer cells, and inhibiting tumor growth in experimental animals. Bax/ Bcl-2、Cleaved-PARP、Caspase-3、LC3-II、p-ERK1/2、p-c-Jun ↑ Bcl-2 ↓	[43]
	Cucurbitaceae	*Bolbostemma paniculatum*	Tubeimoside-1	Prostatic cancer	Cytotoxicity, induction of cancer cell apoptosis, G0/G1 phase arrest, reduction of mitochondrial membrane potential. ROS、p- ASK-1、p-JNK、 p-p38、CHOP、Bax、Cleaved-Caspase-3、p53、p21 ↑ Thioredoxin、Bcl-2、cyclin E、cdk2 ↓	[44]
				Lung cancer	Cytotoxicity, inhibition of cancer cell proliferation, induction of mitochondrial fission, induction of autophagy, inhibition of lysosomal acidification, blocking of autophagic flux, induction of reduced mitochondrial membrane potential, suppression of tumor growth in experimental animals. ROS、LC3-II、p62、cathepsin B、Bax、cytoplasmic cytochrome C、Cleaved-PARP、Cleaved-Caspase-3 ↑ p-DRP1、mature cathepsin D、V-ATPase activity ↓	[45]
				Cervical carcinoma	Cytotoxicity, inhibition of cancer cell proliferation, induction of apoptosis, induction of autophagy, suppression of autophagic flux, inhibition of lysosomal activity, suppression of tumor growth in experimental animals. Cleaved-Caspase-3、Cleaved-PARP、LC3-II、Beclin 1、Atg5、p-AMPK、p-ACC、p62、LAMP1、LAMP2、 RAB5、RAB7 ↑	[46]
				Breast cancer	Activate autophagy and induce apoptosis in cancer cells. LC3-II、p-eEF2、Cleaved-PARP、Cleaved-Caspase-3 ↑ p-Akt(Ser473、Thr308)、p-p70S6K、Mcl-1、Bcl-xL、Bcl-2 ↓	[47]
	Ranunculaceae	*Anemone raddeana*	Raddeanin A	Breast cancer	Enhance autophagy flux, suppress cancer cell viability, and induce apoptosis. LC3-II、p-eEF2、Cleaved-Caspase-3、Cleaved-PARP ↑ p-S6K、p-Akt、Bcl-xL、Mcl-1、Bcl-2 ↓	[48]
				Colorectal cancer	Inhibits cancer cell proliferation and migration, suppresses angiogenesis, and exhibits structural docking with VEGFR2 kinase. p-VEGFR2、p-plc - γ − 1、p-JAK2、p-FAK、p-Src、p-Akt ↓	[49]
					Cytotoxicity, induction of cancer cell apoptosis, reduction of mitochondrial membrane potential, inhibition of cancer cell proliferation, G0/G1 phase arrest, and suppression of tumor growth in experimental animals. Bax、Cleaved-Caspase-3、Cleaved-PARP、p-β-catenin ↑ Bcl-2、c-Myc mRNA、Cyclin D1 mRNA、β-catenin、p-GSK-3β、p-Akt、p- LRP6、p-IKBα ↓	[50]
				Osteosarcoma	Cytotoxicity, inhibition of cancer cell proliferation, induction of apoptosis, suppression of tumor growth in experimental animals. ROS、Cleaved PARP、Cleaved-Caspase- 8、Cleaved-Caspase- 9、Cleaved-Caspase- 3、Bax、p-ERK1/2、p-JNK、p-c- Jun ↑ Bcl- 2、Bcl- xl、p-STAT3 ↓	[51]
					Cytotoxicity, inhibition of cancer cell proliferation, induction of apoptosis. Cleaved-PARP、Bax ↑ Bcl-2、Bcl-xL、p-STAT3、p-JAK2、MDR1 ↓	[52]
				Prostatic cancer	Depends on the androgen receptor (AR) to inhibit cancer cell growth and suppress AR signaling. AR-FL、AR-V and their mRNA ↓	[53]
		*Pulsatilla chinensis*	α-Hederin	Colon cancer	Cytotoxicity, inhibition of cancer cell proliferation, induction of apoptosis, induction of endoplasmic reticulum stress, induction of autophagy, blockade of autophagy flux. Cleaved-PARP、Cleaved-Caspase-3、GRP78、PERK、eIF2α、p-eIF2α、IRE1、ATF6、calnexin、ATF4、Bax、p-IRE1、p-JNK、p-p38、LC3-II、p62 ↑ Bcl-2 ↓	[54]
				Gastric cancer	Inhibits cancer cell proliferation, G1 phase arrest, induces apoptosis, reduces mitochondrial membrane potential, and suppresses tumor growth in experimental animals. P16、P21、P53、cdk2、Bax、Cleaved-Caspase-3、Cleaved-Caspase‐8、Cleaved-Caspase‐9、ROS ↑ Cyclin D1、Bcl-2、glutathione、ATP ↓	[55]
				Ovarian cancer	Inhibits cancer cell growth, induces nuclear fragmentation and chromatin condensation, triggers apoptosis, reduces mitochondrial membrane potential, G0/G1 phase arrest. Caspase-9 activity、Caspase-3/7 activity ↑ Bcl-2↓	[56]
steroidal saponins	Asparagaceae	*Anemarrhena asphodeloides*	Timosaponin AIII	Breast cancer	Induce cancer cell senescence, inhibit cancer cell proliferation, migration, and invasion. miR-141、miR-200c ↑ BMI1 and its mRNA、H2AUb、c-Myc、PRC1 activity ↓	[57]
				Lung cancer	Cytotoxicity, induction of cancer cell apoptosis (at high concentrations), induction of mitochondrial membrane potential reduction, induction of autophagy (at low and high concentrations) with activation of autophagic flux, and inhibition of tumor growth in experimental animals. Cleaved-Caspase-3、Cleaved-Caspase-8、Cleaved-Caspase-9、Cleaved-PARP、cyt C、AIF、EndoG、Bax、LC3-II、Beclin 1、p-AMPK ↑ p-Erk1/2 ↓	[58]
				NSCLC	Inhibits the growth and proliferation of cancer cells both in vivo and in vitro, causing G2/M cycle arrest, suppressing cell migration and invasion, and reducing mitochondrial membrane potential. Forms a complex with HSP90, further targeting and triggering the ubiquitination and degradation of GPX4, thereby inducing ferroptosis in cancer cells. E-cadherin、ROS、HMOX-1、HSP90 ↑ VIM、SNAIL-2、SNAIL-1、MMP-9、FTL、GPX4、SLC40A1、SLC7A11 ↓	[59]
				Pancreatic cancer	Inhibit cancer cell growth, causing G2/M phase arrest, and induce apoptosis. p-JNK(high dose)、p-ERK1/2(low dose)、p21 ↑ ERK1/2、STAT3、p-ERK1/2、p-STAT3、Src、p-Src、p-JNK(low dose)、p-ERK1/2(high dose)、Bcl-2、cyclin D1、MMP-9、VEGF-1 ↓	[60]
	Dioscoreaceae	*Dioscorea tokoro*	Dioscin	Cervical carcinoma	Cytotoxicity, DNA damage induction, apoptosis induction. ROS、Ca^+^、cytoplasmic cytochrome C、Bax、Bak、Bid、p53、Caspase-3、Caspase-9 ↑ Bcl-2、Bcl-xL ↓	[61]
				Laryngocarcinoma	Cytotoxicity, causing S phase arrest, inducing apoptosis, inducing DNA damage, inhibiting cancer cell migration and invasion. ROS、p53、p-JNK、p-p38、cytoplasmic cytochrome C、Bax、Cleaved-Caspase-3、Cleaved-Caspase-9 ↑ MMP-2、MMP-9、CDK2、Cyclin A、p-ERK1/2、Bcl-2 ↓	[62]
				Liver cancer	Cytotoxicity, inhibition of cancer cell proliferation and migration. connexin 43、ZO-1、claudin-1、E-cadherin ↑ MKK3、p-Erk、p-p38、p-JNK、N-cadherin、Vimentin、Snail ↓	[63]
				Lung cancer	Cytotoxicity, inhibition of cancer cell proliferation, suppression of cancer cell migration and invasion, inhibition of EMT, induction of apoptosis, and suppression of tumor growth in experimental animals. E-cadherin、Bax、Cleaved-Caspase-3、Cleaved-PARP、p-p38、p-HSP27、ROS ↑ N-cadherin、Vimentin、Bcl-2 ↓	[64]
				Osteosarcoma	Cytotoxicity, inhibition of cancer cell proliferation, causing G2/M phase arrest, induction of apoptosis, induction of decreased mitochondrial membrane potential, induction of cancer cell pyroptosis, inhibition of tumor growth in experimental animals. P53、P21、p- Cdc2、p-ATM、Cleaved-PARP、Cleaved-Caspase-3、Cleaved-Caspase-8、Cleaved-Caspase-9、cytochrome C、Bax、p-p38、p-JNK、LDH release、GSDME – N ↑ Bcl-2、Ki-67 ↓	[65]
				Prostatic cancer	Cytotoxicity, induction of cancer cell apoptosis, inhibition of proliferation, inhibition of cancer cell migration, inhibition of tumor growth in experimental animals. p-SHP1、Caspase-3、Bad ↑ p-Erk1 /2 (T202/T204)、p-p38 (T182)、p-Akt (T326) ↓	[66]
	Liliaceae	Rhizoma Paridis	Polyphyllin II	Colorectal cancer	Inhibits cancer cell growth, induces apoptosis, causes G1 cycle arrest, suppresses mitochondrial fission, and inhibits p65 nuclear translocation. cyclin D1、c-Myc、p-DRP1、p-ERK、p-IKKα、p-p65 ↓	[67]
				Liver cancer	Inhibits cancer cell activity, induces apoptosis, induces a decrease in mitochondrial membrane potential, enhances mitochondrial membrane permeability, and causes S-phase cycle arrest. ROS、p53、p21、Bax、cytoplasmic cytochrome C、Fas、Cleaved-Caspase-3、 Cleaved-Caspase‐8、Cleaved-Caspase‐9、Cleaved-PARP ↑ cyclin A、cyclin E、CDK 2、Bcl-2、mitochondrial cytochrome C ↓	[68]
				Lung cancer	Induce autophagy and apoptosis in cancer cells. LC3-II、Beclin-1、cytochrome C、Caspase 9、Cleaved-Caspase3、Bax、p-JNK ↑ p62、Bcl-2、p-PI3K、p-AKT、p-mTOR、ROS ↓	[69]
	Melanthiaceae	*Paris polyphylla*	Polyphyllin D	Breast cancer	Cytotoxicity, induction of cancer cell apoptosis, induction of autophagy, enhancement of Beclin1-Vps34 interaction, inhibition of tumor growth in experimental animals. Bax、Cleaved-Caspase-3、Cleaved-Caspase-8、LC3-II、Beclin1、p-JNK、p-Bcl-2 ↑ Bcl-2、p62 ↓	[70]
				Leukemia	Cytotoxicity, induction of cancer cell apoptosis, induction of decreased mitochondrial membrane potential, induction of differentiation of human chronic myeloid leukemia cells into monocytes or mature macrophages. Bax、Cleaved-Caspase-3、CD14 ↑ Bcl-2、p210-Bcr/Abl ↓	[71]

* “↑” and “↓” in the table indicate up and down regulation, respectivel

The research results demonstrated that saponins with clear anti-tumor effects mostly have cytotoxicity and can exert significant anti-cancer effects by inhibiting cancer cell proliferation, inducing cell apoptosis, inhibiting cell migration, and inducing cell cycle arrest. In addition, some saponins can also exhibit remarkable anti-tumor effects in both in vitro and in vivo experiments on various cancers by such as promoting cellular autophagy, inducing DNA damage, inducing cell iron death, inhibiting glycolysis, inhibiting epithelial-mesenchymal transition (EMT), inhibiting angiogenesis, and regulating protein-protein interactions.

## Combination of saponins and chemotherapeutic drugs

Given the well-characterized antitumor mechanisms of saponin monomers elaborated above, and in view of the clinical predicament of chemotherapeutic drugs accompanied by severe side effects, drug resistance and other limitations. Some articles categorize saponins by their use in combination with different chemotherapeutic agents, focusing on examining the synergistic effects and reduction in toxicity achieved by these combination therapies [72]. In recent years, studies [73–75] have found that the combined use of saponin components from natural products and chemotherapeutic drugs can generate a multi-dimensional synergistic effect. It can demonstrate the unique advantages of the combined medication in four aspects: synergistic enhancement of efficacy, reduction of toxicity and protection, reversal of drug resistance, and improvement of immunity, thus providing new ideas for optimizing tumor treatment strategies. This section is outcome-oriented, categorizing the various effects of combination therapy to facilitate a clearer understanding of its advantages and potential for development in cancer treatment.

### Combined application to improve the efficacy of chemotherapy drugs

**Fig. 2:**
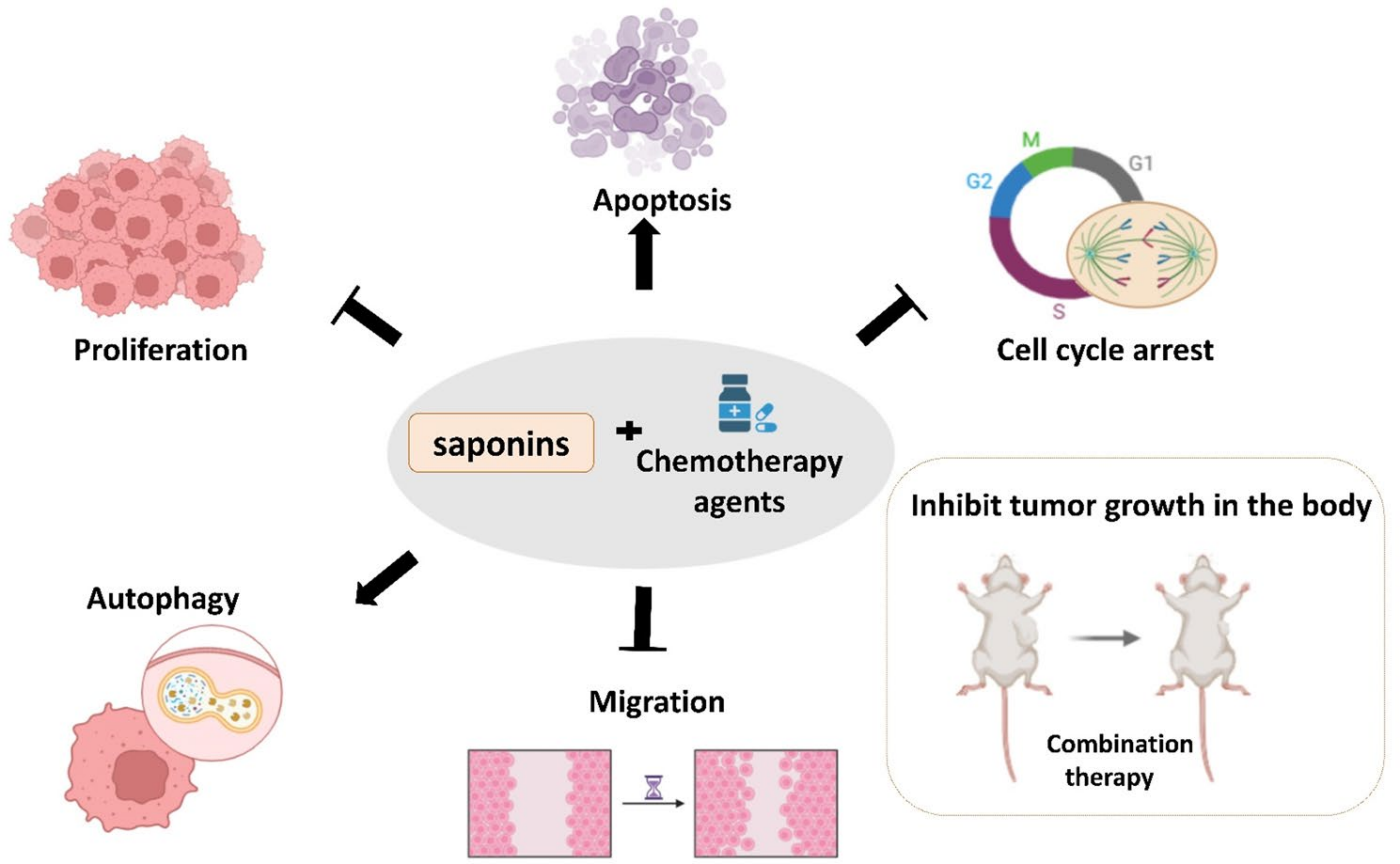
Saponins enhance the efficacy of chemotherapeutic drugs through multiple pathways

Several studies have revealed the synergistic anti-tumor potential of natural active ingredients combined with conventional chemotherapy drugs, which improves anti-tumor efficacy by regulating apoptosis, autophagy, cell cycle, oxidative stress, and key signaling pathways (Fig. 2). Through in vitro cell models and in vivo animal experiments, these researches elucidate how drug combinations enhance the efficacy of chemotherapy drugs through different molecular mechanisms, providing a theoretical basis for optimizing clinical treatment.

An experimental study [76] demonstrated that the triterpenoid saponin α-Hederin, when combined with cisplatin—a commonly used clinical chemotherapeutic agent—synergistically enhanced the inhibitory effect on gastric cancer cells in a time- and concentration-dependent manner across multiple gastric cancer cell lines. Meanwhile, this combination regimen could increase the accumulation of reactive oxygen species (ROS), upregulate the expression of apoptosis-related factors, and reduce the mitochondrial membrane potential (MMP). Consequently, it inhibited the proliferation and migration of cancer cells and promoted their apoptosis. At the same time, in the xenograft mouse model, the combined use of the two drugs exerted a significantly more potent tumor-suppressive effect than either drug alone.

Triterpenoid saponins CIL 1/2, extracted from *Lysimachia ciliata*, exhibited no cytotoxicity towards prostate cancer cells (DU-145 and PC-3) and normal prostate cells at a concentration of 0.5 µg/mL. When combined with 0.05 µg/mL mitoxantrone, it exerted synergistic cytostatic and proapoptotic effects on prostate cancer cells, synergistically inhibiting their migration and invasion. Concurrently, the combined treatment exhibited significantly lower cytotoxicity towards normal cells compared to mitoxantrone alone [77].

The combination of 20(S)-ginsenoside Rg3 and sorafenib significantly inhibits hepatocellular carcinoma cell proliferation and induces apoptosis by modulating the PTEN/Akt signaling pathway. In mouse models, the combination therapy group showed significantly reduced tumor volume and weight compared to the monotherapy groups [78]. Another study confirmed that 20(S)-ginsenoside Rg3 and sorafenib exert synergistic antitumor effects by regulating HK2-mediated glycolysis and the PI3K/Akt signaling pathway [79].

The combination of ginsenoside CK and cytarabine in the treatment of acute myeloid leukemia (AML) showed a synergistic effect in four AML cell lines (THP-1, U937, HL-60 and MOLM-13). With the increase in the concentration of ginsenoside CK, the half maximal inhibitory concentration (IC_50_) value of cytarabine decreased gradually. Moreover, ginsenoside CK can promote cytarabine-induced lactate dehydrogenase (LDH) release, mitochondrial dysfunction, and DNA damage in THP-1 and U937 cells, thereby promoting cell apoptosis and exerting a stronger anti-leukemia effect [80].

Saikosaponin A can selectively target quiescent cancer cells (QCC) and enhance autophagy of QCC by inhibiting Akt/mTOR pathway, thus reversing the increase of QCC caused by docetaxel. Using saikosaponin A during the intervals between docetaxel treatment cycles can induce QCC death in vitro and reduce tumor volume and weight in mouse models, thus avoiding treatment failure and cancer recurrence. Meanwhile, weight loss was reduced in the combination group compared with the docetaxel group [81].

Steroidal saponin polyphyllin I (PPI) can induce apoptosis through caspase-dependent pathway and activate autophagy through PI3K/Akt/mTOR pathway. When combined with autophagy inhibitor chloroquine, PPI-induced autophagy is inhibited, and PPI-induced apoptosis is enhanced, which significantly inhibits cell proliferation compared with single drug application [82].

The combination of the steroidal saponin DT-13 and vinorelbine demonstrates a synergistic effect. DT-13 enhances vinorelbine-mediated mitotic arrest by suppressing the expression of FOXM1 and BICD2. This mechanism potentiates the inhibition of cell proliferation, induces apoptosis, and promotes cell cycle arrest. Consistent with these effects, combination treatment significantly reduced tumor volume compared with the two monotherapy groups in nude mouse xenograft models [83].

In summary, all the aforementioned saponin-drug combination cases share commonalities in their core mechanisms, yet exhibit differences in their specific action mechanisms and application scenarios. These commonalities are manifested in two aspects: First, all combinations demonstrate synergistic anticancer effects in either in vivo or in vitro experiments, with efficacy significantly superior to that of monotherapy with chemotherapeutic drugs, thereby providing reliable experimental evidence for their potential clinical application. Second, the core targets of their synergistic actions focus on inhibiting cancer cell proliferation and promoting apoptosis, which are critical steps in suppressing tumor progression. The primary differences are reflected in two dimensions: First, the synergistic mechanisms vary when different types of saponins are paired with corresponding chemotherapeutic agents to target distinct tumor types. These mechanisms include regulating reactive oxygen species (ROS) accumulation and mitochondrial function, targeting specific signaling pathways (e.g., PTEN/Akt, Akt/mTOR, PI3K/Akt/mTOR), modulating autophagy, concentration-dependently enhancing the sensitivity of chemotherapeutic drugs, and targeting quiescent cancer cells (QCC) or mitotic processes. Second, each combination offers unique advantages: CIL 1/2 reduces the toxicity of chemotherapeutic agents to normal cells, while saikosaponin A prevents tumor recurrence by targeting QCC. Additionally, some combinations (e.g., ginsenoside CK) allow for flexible dose adjustments. Collectively, these commonalities and differences validate the broad application prospects of saponin-drug combinations in cancer treatment, highlighting the importance of selecting appropriate combinations based on tumor type and therapeutic needs.

### Combined application to reduce the toxic and side effects of chemotherapy drugs

**Fig. 3:**
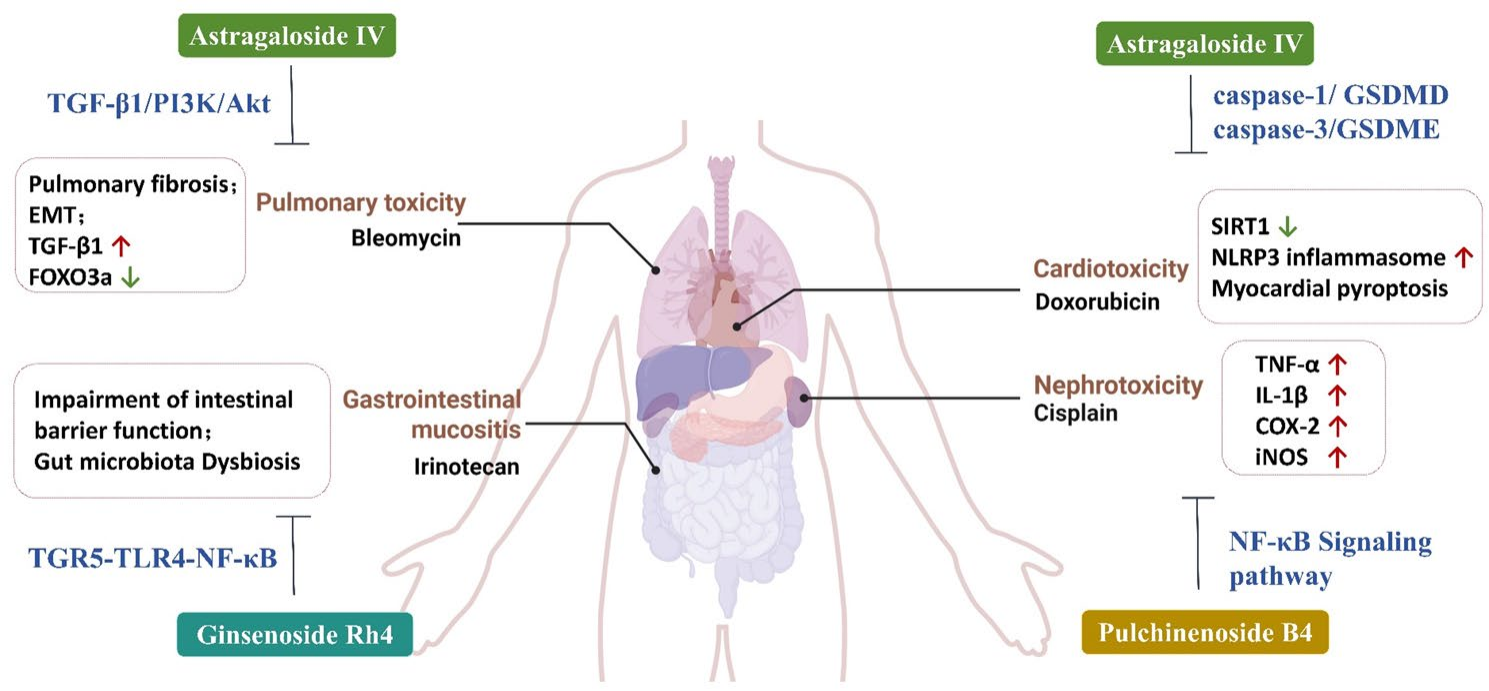
Saponins can improve the toxic and side effects caused by various chemotherapeutic drugs

With the widespread application of chemotherapy drugs, reducing the toxic and side effects of chemotherapy drugs has become a key problem in improving patients’ quality of life. Many studies have investigated the protective effect of natural active ingredients against chemotherapy-related toxicity, revealing that they can effectively alleviate the damage of drugs to organs such as the kidney, heart, and intestine by regulating oxidative stress, inflammatory response, and key signaling pathways. This thereby shows the multi-target intervention mechanism of natural ingredients in organ protection and reflects the potential of saponins in enhancing the safety of cancer chemotherapy (Fig. 3).

The triterpenoid saponin pulchinenoside B4 ameliorates cisplatin-induced acute kidney injury (AKI). Co-administration of pulchinenoside B4 (150 and 300 mg/kg) with cisplatin elevates the spleen index, reduces the kidney index, and effectively normalizes it. Concurrently, this combination significantly decreases serum levels of the nephrotoxicity markers, blood urea nitrogen and creatinine, compared to the cisplatin-only control group. H&E staining revealed that the combination therapy alleviated cisplatin-induced renal damage, manifested as reduced tubular congestion and epithelial cell atrophy, which resulted in lower histopathological scores. By inhibiting the NF-κB signaling pathway, pulchinenoside B4 reduces serum and renal levels of TNF-α and IL-1β, suppresses renal expression of COX-2 and iNOS, and counteracts cisplatin-induced inflammatory responses. These effects collectively demonstrate its anti-inflammatory activity and provide renal protection [84].

Astragaloside IV significantly alleviated doxorubicin-induced atrophy of glomerulus, tubular lumen dilatation, and periglomerular fibrosis, as well as reduced the elevated levels of blood urea nitrogen and serum creatinine in mice. It can improve renal dysfunction, reduce oxidative stress, alleviate iron accumulation, and inhibit doxorubicin-induced ferroptosis of renal cells by activating the PI3K/Akt and Nrf2 signaling pathways [85]. Astragaloside IV combination therapy markedly attenuated doxorubicin-induced cardiotoxicity. It improved cardiac function (ejection and contraction fractions), reduced serum levels of injury markers (troponin MB and LDH), and reversed adverse structural remodeling by decreasing left ventricular end-systolic diameter and increasing posterior wall thickness. Furthermore, hematoxylin and eosin (H&E) staining revealed markedly attenuated myocardial injury in the combination therapy group compared to the doxorubicin-only group. Astragaloside IV can reverse the downregulation of SIRT1 expression in cardiac tissue after doxorubicin treatment and thus inhibit the formation of NLRP3 inflammasome through caspase-1/GSDMD and caspase-3/GSDME pathways, reduce myocardial pyroptosis, and reduce cardiotoxicity [86]. Astragaloside IV can also reversed bleomycin-induced weight loss in rats and reduced the lung wet-to-dry weight ratio, indicating alleviation of pulmonary edema. Furthermore, histological analysis demonstrated that astragaloside IV treatment ameliorated diffuse pulmonary fibrosis and significantly reduced collagen deposition. Mechanistically, astragaloside IV exerts its protective effect against pulmonary fibrosis by activating FOXO3a through inhibition of the TGF-β1/PI3K/Akt signaling pathway, thereby preventing EMT in bleomycin-induced models [87].

Ginsenoside Rh4 repairs intestinal barrier function damage induced by irinotecan (CPT-11) and restores intestinal mucosal homeostasis in an intestinal microbiota-dependent manner. Combined treatment with Ginsenoside Rh4 ameliorated CPT-11-induced weight loss, diarrhea, colon shortening, and abnormal increases in intestinal permeability in mice. Histochemical staining showed that Rh4 combination therapy reduced histopathological damage. Furthermore, Rh4 significantly decreased the expression levels of pro-inflammatory cytokines, including TNF-α, IL-1β, and IL-6, thereby alleviating CPT-11-induced gastrointestinal mucositis. It also improved the diversity of the intestinal microbiota and counteracted CPT-11-induced microbial dysbiosis. Further studies have shown that these protective effects are mediated through the TGR5-TLR4-NF-κB signaling pathway [88].

Pre-treatment with the steroidal saponin dioscin improved cisplatin-induced intestinal villus disruption and inflammatory cell infiltration, promoted proliferation of goblet cells in intestinal crypts, and reduced serum diamine oxidase levels. The histopathological injury score in the combination therapy group was significantly lower than that in the cisplatin group. Furthermore, the combination therapy significantly reduced NLRP3 inflammasome formation in ileal epithelium, thereby mitigating cisplatin-induced intestinal toxicity through alleviation of oxidative stress and inflammatory responses [89].

All of the aforementioned saponins effectively alleviate organ damage induced by their corresponding chemotherapeutic agents (e.g., cisplatin, doxorubicin, and CPT-11), as evidenced by improved organ function, attenuated histopathological injury, and decreased levels of relevant damage markers. Furthermore, their core protective mechanisms focus on suppressing inflammatory responses, alleviating oxidative stress, and regulating related signaling pathways—all critical processes in counteracting chemotherapy-related toxicity. Third, in vivo experiments have validated these protective effects, providing reliable experimental evidence for mitigating chemotherapy-induced side effects and enhancing the body’s tolerance to chemotherapy.

Notably, different saponins exert protective effects through distinct mechanisms and signaling pathways: Astragaloside IV effectively counteracts ferroptosis, pyroptosis, and fibrosis by regulating multiple signaling pathways, including PI3K/Akt, Nrf2, SIRT1-NLRP3, and TGF-β1/PI3K/Akt; Ginsenoside Rh4 restores intestinal barrier function by modulating the gut microbiota and the TGR5-TLR4-NF-κB pathway; while diosgenin alleviates chemotherapy-induced intestinal toxicity by inhibiting NLRP3 inflammasome assembly and reducing oxidative stress.

It warrants consideration whether the same saponin can exert consistent protective effects when mitigating similar side effects induced by different chemotherapeutic agents. For instance, studies have demonstrated that pulchinenoside B4 ameliorates cisplatin-induced renal injury, but its efficacy in alleviating doxorubicin- or other chemotherapy-induced renal damage remains inconclusive. This discrepancy may stem from the differing mechanisms by which these chemotherapeutic agents induce the side effect.

Overall, these studies demonstrate that saponins hold considerable potential for preventing chemotherapy-induced organ toxicity. However, their clinical application and research require precise matching based on specific chemotherapeutic agents and the target organs affected by their toxicity, underscoring their profound research significance and practical value.

### Reversal of chemotherapeutic drug resistance by combined application

**Fig. 4:**
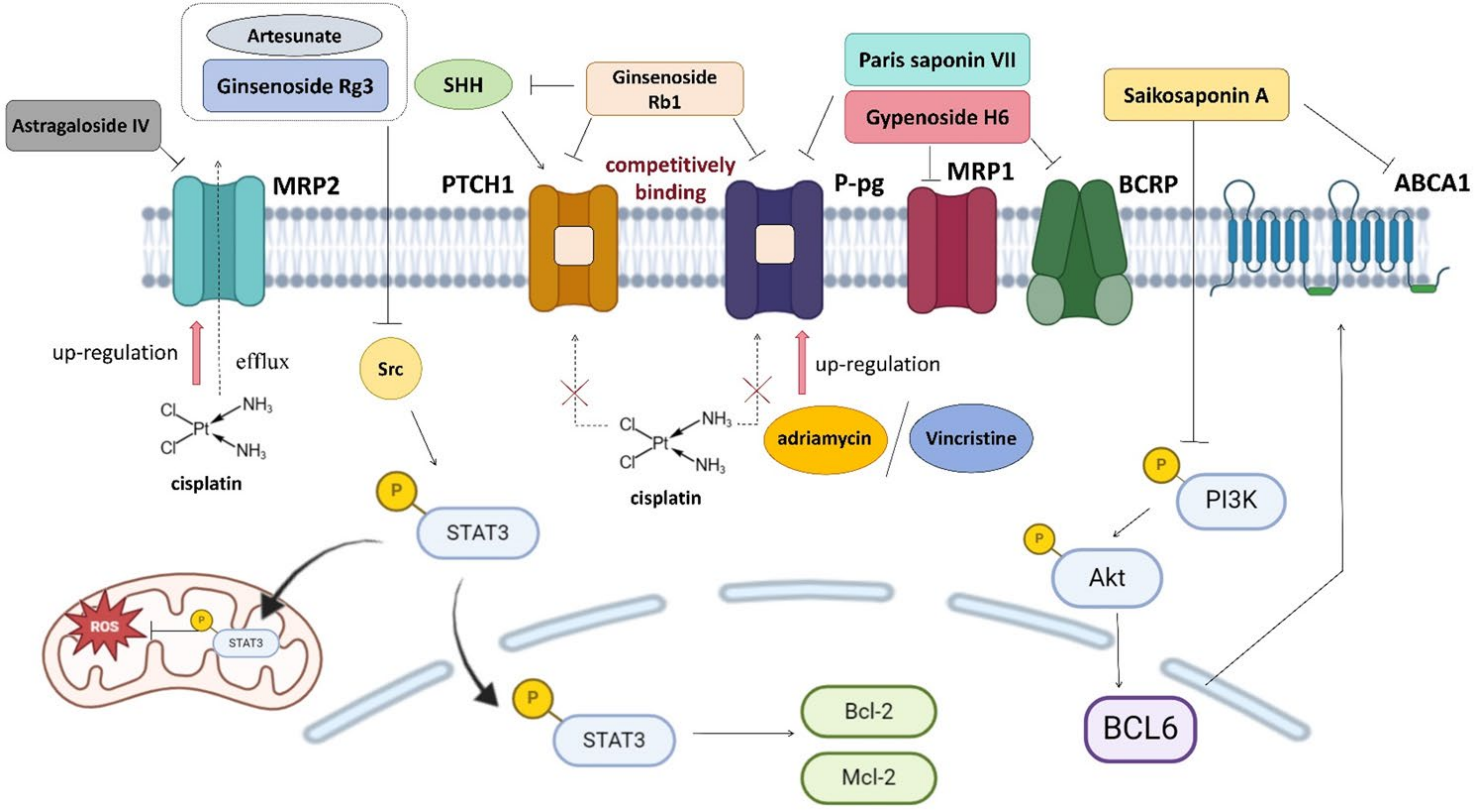
The combination of saponins and chemotherapeutic drugs can reverse drug resistance through multiple pathways

Resistance to chemotherapy drugs is a major obstacle in cancer treatment. Natural compounds can significantly enhance the sensitivity of tumor cells to chemotherapeutic drugs. They do this by inhibiting drug efflux pumps, blocking protective autophagy, and regulating drug-resistance-related signaling pathways (Fig. 4).

The combination of 40 µM astragaloside IV and 2 µM cisplatin (neither concentration exhibiting significant toxicity to HepG2 cells) significantly decreased the cell viability, induce tumor cell apoptosis, slow down the growth of tumor cells both in vitro and in vivo, and improve the chemosensitivity of liver cancer cells to cisplatin by reversing the overexpression of multidrug resistance-associated protein MRP2 in tumor cells induced by cisplatin. Thus, it can inhibit the efflux of cisplatin and enhance the drug sensitivity [90].

The triterpenoid saponin tubeimoside-I itself has excellent anti-tumor activity by inhibiting cell proliferation, inducing cell apoptosis, and suppressing autophagy flux. At the same time, it can be used as specific autophagy flux inhibitor. When combined with 5-FU/doxorubicin, it significantly inhibits the activity and proliferation of two types of colon cancer cells, improving their sensitivity to chemotherapy drugs [91].

The combination of ginsenoside Rg3, artesunate and sorafenib enhances the inhibitory effect on sorafenib-resistant hepatocellular carcinoma cells and reduces the resistance of hepatocellular carcinoma to sorafenib. These effects are achieved by inhibiting the activation/phosphorylation of Src and STAT3 (which are related to sorafenib resistance) in hepatocellular carcinoma cells or tumors, reducing the nuclear levels of STAT3, and inducing the production of ROS [92].

Ginsenoside Rb1 can effectively reverse the in vitro and in vivo resistance of A549/DDP cells to cisplatin. After the combination of the two drugs, it can inhibit the function of two drug pumps (P-gp and PTCH1) by targeting the ABCB1 and Hedgehog pathways, increase the intracellular concentration of cisplatin, and improve the sensitivity of cancer cells to cisplatin. It is accompanied by the downregulation of the expression levels of ABCB1, SHH, PTCH1, and GLI2 mRNA and proteins, and promotes cell apoptosis through the relative regulation of the expression levels of Bax and Bcl-2 proteins [93].

After treating MCF-7/ADR cells (an adriamycin-resistant cell line) with low-dose Paris saponin VII and adriamycin at different concentrations (0–100 µM), the combination enhanced the intracellular adriamycin fluorescence signal, significantly reduced the IC₅₀ value of adriamycin in drug-resistant cells, and promoted apoptosis. It also reversed the overexpression of P-gp in drug-resistant cells. Compared to the monotherapy group, combination treatment significantly reduced tumor size in nude mice bearing MCF-7/ADR cell xenografts, and the experimental dose caused no significant weight loss or toxicity signs [94].

The combination of gypenoside H6 and vincristine has a significant synergistic effect. gypenoside H6 can reverse potential multidrug resistance by inhibiting the expression of glycoprotein P-gp, multidrug resistance related protein (MRP1) and breast cancer resistance protein (BCRP) [95].

Saikosaponin A can enhance chemosensitivity to gemcitabine by targeting the p-Akt/BCL6 signaling pathway in the treatment of intrahepatic cholangiocarcinoma, ultimately reducing the expression of the predictive biomarker ABCA1 for drug resistance [96].

In summary, the aforementioned studies consistently demonstrate that multiple saponins can effectively reverse chemotherapy resistance in various cancer types and enhance chemosensitivity, through diverse mechanisms of action. Although ginsenoside Rb1, paris saponin VII, and gypenoside H6 primarily target classic efflux transporters, their major additional mechanisms differ: Rb1 regulates the Hedgehog signaling pathway, paris saponin VII suppresses P-gp expression, and gypenoside H6 also modulates BCRP. Collectively, these findings indicate that inhibiting drug efflux constitutes a key strategy for overcoming chemotherapy resistance. Moreover, the distinct secondary signaling pathways regulated by different saponins support their precise application against specific resistance mechanisms in particular cancer types.

### Combination application may improve the immune function of chemotherapy patients

**Fig. 5:**
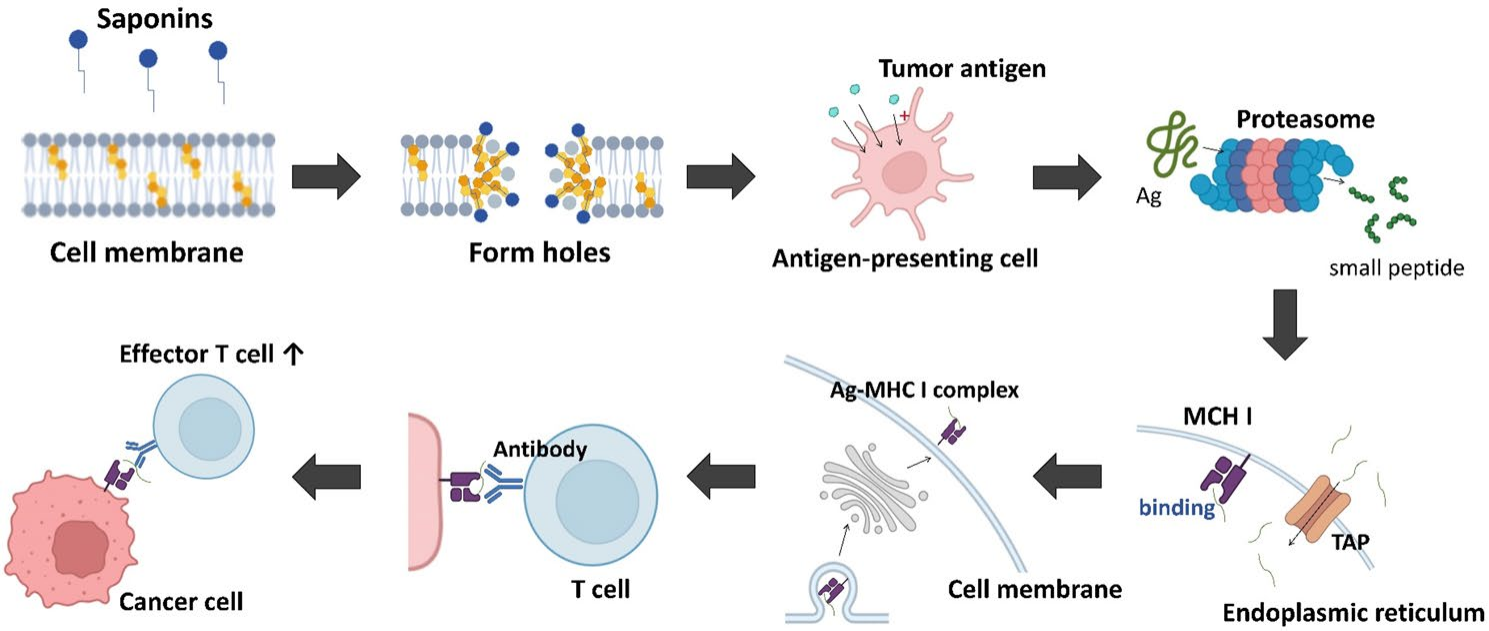
Saponins can enhance the immune function of the body by “forming holes” in the cell membrane

Because saponin components can create pores in the cell membrane, allowing antigens to enter the cell and be presented by the major histocompatibility complex MHC I, ultimately increasing the number of effector T cells (Teffs) [97], thus having the function of enhancing immunity (Fig. 5). Simultaneously, certain saponins can induce immunogenic cell death (ICD) in cancer cells by acting on proteins such as calreticulin, heat shock proteins, and high-mobility group box 1 (HMGB1). This transforms the cells from a non-immunogenic state to an immunogenic state, thereby mediating the body’s antitumor immune response. Alternatively, they may inhibit tumor growth by regulating immune factors [97]. Studies have shown that some saponin components, such as ginsenoside Rg3 [98] and gypenoside [99], can regulate the function of immune cells.

Currently, they mainly serve as vaccine adjuvants and play wide roles in the field of anti-tumor treatment [100]. Among them, QS-21, a triterpenoid saponin from *Quillaja saponaria* Molina, is the most well-known. Because of its low toxicity and ability to enhance antibody responses and promote specific T cell responses, it serves widely as a vaccine adjuvant [101], and many clinical trials have demonstrated its favorable immune adjuvant properties in the treatment of melanoma, ovarian cancer, breast cancer and other cancers [102]. Anemoside B4 can have a positive effect on the immune response by increasing the number of lymphocytes and enhancing the activity of lymphocytes. It exhibits immune-adjuvant-like properties in mouse models, stimulating the growth and development of the spleen and thymus. Meanwhile, it can induce a significant enhancement of Th1 (IgG2a, IgG2b, IL-2, IFN-γ) and Th2 (IL-4, IL-10, IgG1) immune responses [103]. Raddeanin A—a triterpenoid saponin isolated from sea anemones—exerts direct cytotoxicity on tumor cells by triggering ICD, and further promotes the activation of tumor-infiltrating CD8 + T cells and their secretion of IFN-γ and GZMB, thereby promoting anti-tumor immunity. This key mechanism forms a bridge between direct cytotoxicity and immune activation [104]. Gypenoside can enhance the antitumor function of T cells against gastric cancer cells by inhibiting the expression of PD-L1 in these cells, meanwhile inhibiting tumor growth and promoting apoptosis [99]. The steroidal saponin taccaoside A enhances the mTORC1-Blimp-1 signaling pathway to promote GZMB secretion, thereby regulating the immune response of T lymphocytes against various tumor cells, and demonstrates anti-tumor efficacy in vivo [105].

Some chemotherapy drugs, while killing rapidly proliferating tumor cells, may cause damage to the function of immune cells [106], reshape the immunosuppressive microenvironment [107], and disrupt the structure and function of immune organs [108], thereby significantly inhibiting the body’s immune system. Combining them with saponins may overcome the limitations of chemotherapy drugs with regard to immune function. Cyclophosphamide (CTX) is a clinically widely used broad-spectrum anticancer drug, with immunosuppression as its primary side effect. Ginsenosides can alleviate CTX-induced bone marrow suppression by improving bone marrow function, thereby restoring hematopoietic function and immunity [109]. Among these, different doses of ginsenoside Rb2 alleviated CTX-induced decreases in body weight and spleen index in mice, the latter being a key indicator reflecting splenic structural integrity and proliferative activity. Hematoxylin and eosin (H&E) staining confirmed that Rb2 ameliorated damage to the splenic capsule and germinal centers: the splenic capsule preserves the structural framework of the spleen for immune cell lodging and retention, while germinal centers serve as the primary site for immune cell activation and expansion [110, 111]. Repair of these structures re-establishes the normal immune microenvironment of the spleen, thus laying a critical structural foundation for the recovery of splenic immune function. Based on this structural restoration, Rb2 enhanced the proliferation index of splenic lymphocytes, natural killer (NK) cell activity, and phagocytic index α, thereby effectively restoring the numbers and core effector functions of key immune cells. Meanwhile, Rb2 promoted the secretion of pro-inflammatory cytokines including TNF-α and IL-2, and upregulated their corresponding mRNA expression levels, thereby reversing CTX-induced immunosuppression [112]. Total saponins of Panax notoginseng can alleviate both non-specific and specific immune damage induced by CTX, enhance the phagocytic function of mononuclear macrophages, stimulate the proliferation of splenic lymphocytes, promote the release of TNF-α and IL-2, and increase the production of serum hemolysin antibodies. Additionally, they reverse CTX-induced reductions in white blood cells, lymphocytes, and red blood cells, and improve the anti-tumor efficacy of CTX in the treatment of liver cancer. These effects were demonstrated by a significant reduction in tumor volume and weight, as well as improved survival rates in tumor-bearing mice [113].

Although studies have demonstrated that saponins such as QS-21 and anemoside B4 exert potent immunostimulatory effects—including activating T cell responses, enhancing antigen presentation, and boosting antitumor immunity—systematic studies investigating the combination of saponins with chemotherapeutic agents in tumor-bearing models to simultaneously eradicate cancer cells and reverse chemotherapy-induced immunosuppression remain extremely scarce. Existing studies predominantly use cyclophosphamide to establish non-tumor-bearing immunocompromised mouse models, and only verify the restorative effects of saponins on immune organs, immune cells, and cytokine production [114–116]. Notably, few studies have explored combined “chemotherapy plus saponin” regimens in tumor-bearing animals, leaving it unclear whether such strategies can simultaneously achieve tumor suppression and immune reconstitution. This critical knowledge gap hinders the translation of the immunopotentiating potential of saponins into clinical combination chemotherapy regimens. Future studies are urgently warranted to employ tumor-bearing models to determine whether saponins can counteract the immunosuppressive side effects of chemotherapeutic agents such as cyclophosphamide and synergistically enhance antitumor immune responses. Such evidence would provide a solid foundation for the development of more effective and less toxic combinatorial chemotherapy strategies.

### Discussion

The aforementioned studies indicate that when saponins are combined with chemotherapeutic agents, they produce multidimensional synergistic effects, thereby overcoming key limitations of conventional chemotherapy. Such combination therapies exert their effects not through a single mechanism, but by simultaneously modulating multiple core biological processes to enhance therapeutic outcomes.

Within this context, the PI3K/Akt pathway emerges as a central and frequently modulated node. This pathway is involved in enhancing therapeutic efficacy (e.g., 20(S)-Rg3 combined with sorafenib for hepatocellular carcinoma), reducing toxicity (Astragaloside IV counteracts doxorubicin-induced nephrotoxicity), and reversing drug resistance (Saikosaponin A combined with gemcitabine for intrahepatic cholangiocarcinoma). This indicates that saponins can both exploit cancer cells’ dependence on this survival pathway and, in doing so, protect normal tissues. Similarly, modulating NF-κB-mediated inflammatory responses is a common mechanism for mitigating chemotherapy-induced organ damage, exemplified by pulchinenoside B4’s protection against cisplatin-induced nephrotoxicity and dioscin’s alleviation of intestinal toxicity.

Another emerging paradigm involves the dual roles in autophagy regulation. Depending on the context, saponins can either enhance protective autophagy in cancer cells to promote cell death (e.g., saikosaponin A targeting QCC) or inhibit autophagy to overcome drug resistance (e.g., tubeimoside-I combined with 5-fluorouracil/doxorubicin for colon cancer). This duality underscores that autophagy modulation should not be viewed as uniformly beneficial or harmful; therapeutic strategies must align with specific mechanisms.

Furthermore, multiple studies have demonstrated that saponins target drug efflux pumps (P-gp, MRP1, BCRP). Research on ginsenoside Rb1, paris saponin VII, and gypenoside H6 indicates that inhibiting drug efflux represents a primary and effective strategy for reversing multidrug resistance, applicable across various cancer types.

However, critical gaps exist in the current literature. While the immunomodulatory potential of saponins is well-documented—particularly their capacity as vaccine adjuvants to enhance antigen presentation and T-cell responses—their application within combination therapy frameworks to counter chemotherapy-induced immunosuppression remains under-explored. Although a few studies using cyclophosphamide-induced immunosuppression models provide proof-of-concept, these have primarily been conducted in non-tumor animal models. This limits our understanding of whether immune reconstitution can be achieved concurrently with effective tumor control. Future research should prioritize tumor models to determine whether saponins can simultaneously enhance chemotherapy’s direct cytotoxic effects and rebuild antitumor immunity, thereby offering new strategies for preventing recurrence and improving long-term survival rates.

Despite these encouraging findings, limitations remain: most studies are confined to in vitro cell models and in vivo animal experiments, with insufficient clinical evidence; the mechanisms by which saponins modulate the immune system in combination with chemotherapy require further validation in tumor models; and the consistency of their toxicity-alleviating effects across different chemotherapy drugs remains unclear. Future research should focus on these gaps to advance the clinical translation of saponin-chemotherapy combination strategies.

## Saponin-containing drugs for clinical cancer adjuvant therapy

On the basis of clarifying the antitumor effects of saponin monomers and the synergistic value of their combination with chemotherapeutic drugs, this section shifts the focus to clinical translation. At present, some drugs containing saponins or total saponin extracts are used for clinical cancer patients in the Chinese pharmaceutical market. However, these drugs are primarily used to assist chemotherapy or to alleviate post-chemotherapy side effects, rather than being used as direct cancer therapeutics, and all of the following drugs are prescription drugs.

Shenyi capsule is a class I monomer anti-tumour drug in China [117], officially approved by the former National Medical Products Administration (NMPA). Its active ingredient is ginsenoside Rg3 (a tetracyclic triterpenoid saponin), which exerts inhibitory effects on the growth of solid tumors. At the same time, it can suppress the proliferation and growth of tumor vascular endothelial cells and the formation of new blood vessels. It is usually used in combination with chemotherapy drugs to improve the chemotherapy efficacy for primary liver cancer and lung cancer [118]. Furthermore, the results of clinical data analysis show [119] that Shenyi Capsule combined with radiotherapy and chemotherapy can effectively increase the treatment response rate of non-small cell lung cancer (NSCLC), reduce the incidence of leukopenia, enhance the body’s immune response to tumors and its resistance to other pathogenic factors, and significantly improve the Karnofsky Performance Status scores of cancer patients, with stable improvement rates and extended survival periods, thus improving the quality of life of tumor patients.

Kang’ai Injection is a representative modern antitumor preparation derived from traditional Chinese medicine. According to chemical composition analysis, its main active ingredients are ginsenosides, flavonoids and matrine [120]. It is used for the treatment of primary liver cancer, lung cancer, rectal cancer, malignant lymphoma, gynecological malignancies, as well as adjuvant treatment for radiotherapy and chemotherapy [121]. In a clinical study [122], Kang’ai Injection combined with cisplatin-paclitaxel chemotherapy significantly improved the objective response rate and disease control rate in advanced ovarian cancer after 4 cycles. The follow-up results after 2 years showed that the median survival time, progression-free survival time, and cumulative survival rate of the combination group were all significantly longer than those of the chemotherapy group. Moreover, the two tumor markers, CA125 and HE4, decreased more significantly in the combination group, while the indicators of CD3+, CD4+, CD8+, CD16+, and CD56 + in the combination group were all higher than those in the chemotherapy group. This indicates that Kang’ai Injection can enhance the killing effect of chemotherapy drugs on ovarian cancer and, at the same time, reduce the immune damage caused by chemotherapy drugs.

Gypenosides Tablets are an oral solid dosage formulation in which total gypenosides, derived from *Gynostemma pentaphyllum* (Thunb.) Makino, serve as the principal active pharmaceutical ingredient. Although studies [17, 123] have demonstrated its inhibitory effects on liver cancer, lung cancer, and other malignancies, regulators have currently approved them only for the treatment of hyperlipidemia [124], not for primary or adjuvant cancer therapy.

Saponins exhibit potent antitumour activity and immunomodulatory effects, and can mitigate chemotherapy-induced toxicity. Although currently used only as adjunctive agents to alleviate post-chemotherapy side effects, they are anticipated to play a more significant role in future cancer treatment protocols. Advancing their clinical application requires further mechanistic research, optimisation of delivery regimens, and evaluation of long-term safety—particularly in relation to bioavailability and targeted delivery.

## Restrictions and solutions for saponins in antitumor therapy

Although the antitumor activity and clinical application potential of saponins have been demonstrated, their further clinical translation and application remain constrained by inherent limitations such as low bioavailability and hemolytic toxicity. This section summarizes effective improvement strategies targeting these core bottlenecks, elucidating the optimization effects of different methods on the pharmacokinetic properties and safety of saponins, as well as the underlying mechanisms thereof.

### Low bioavailability

**Fig. 6:**
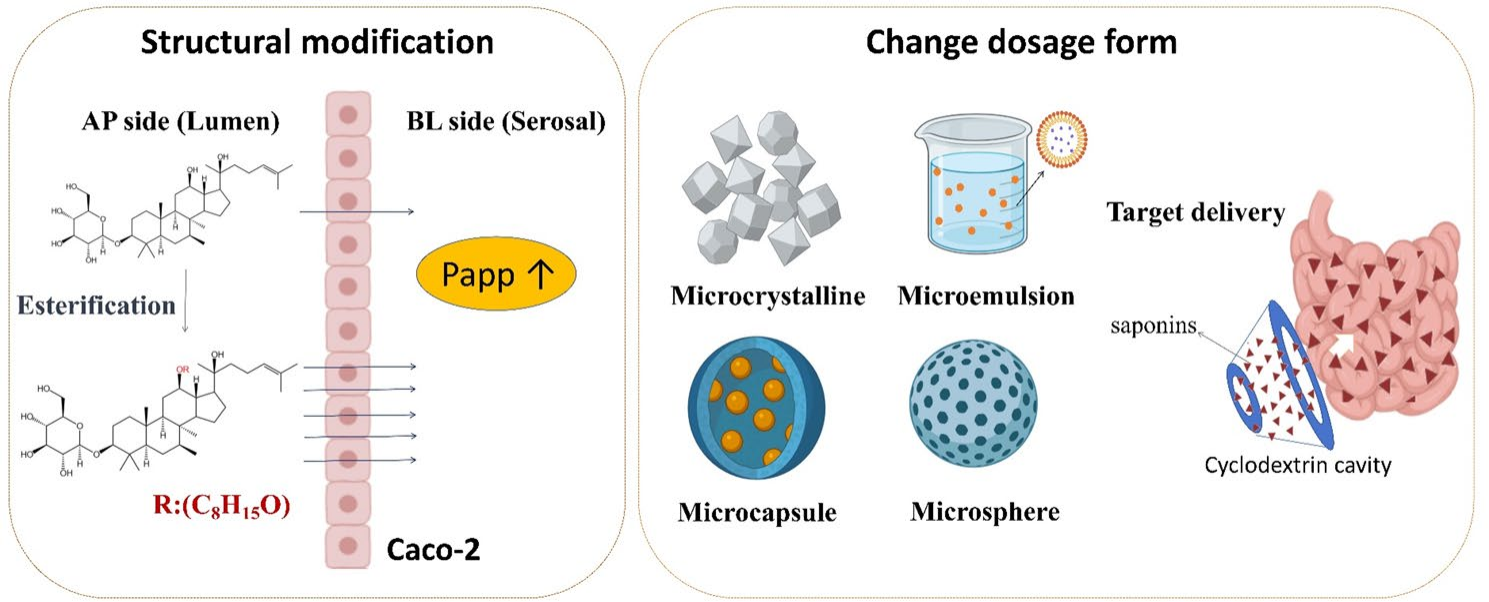
Strategies for improving bioavailability

The molecular mass, flexibility, chemical stability, and amphiphilic properties of saponins themselves significantly affect their ability to overcome various progressive barriers. After ingestion, these barriers include gastrointestinal digestion, microbiota-mediated metabolism, cell permeability, and intracellular metabolic processes [125]. Typically, oral formulations of saponin-based drugs exhibit low bioavailability. This is essentially due to the interaction between the molecular characteristics of saponins and physiological barriers. This problem can be addressed by using saponin derivatives or altering the dosage form to enhance the delivery efficiency (Fig. 6).

The derivatives of saponins mainly include various forms such as esterification, complex formation, and biotransformation. These modification methods can change the physical and chemical properties of saponins, and thereby enhance their bioavailability. Currently, researchers have conducted extensive studies on ginsenosides. Among them, ginsenoside Rh2 exhibits potent antitumor activity against multiple types of cancers. However, due to its high hydrophilicity, it has a relatively low bioavailability, which restricts its development in medical applications.

Compared with ginsenoside Rh2, its derivative Rh2 octyl ester (Rh2-O) increases the apparent permeability coefficient (PAPP) of the transport in the Apical-Basolateral (AP-BL) direction between Caco-2 monolayer cells from 0.21 × 10⁻⁶ cm/s to 1.96 × 10⁻⁶ cm/s [126]. In Hep G2 cells, Rh2-O shows a higher cellular uptake rate than Rh2 [127], thus improving the intestinal absorption and oral bioavailability of the compound. The esterification product derived from the reaction of maleimidocaproic acid with the hydroxyl group at the C-3 glucose position of ginsenoside Rh2 exhibits approximately a 4-fold increase in solubility and enhanced inhibitory activity against the HeLa cell line [128]. When ginsenoside binds to alpha-lactalbumin (ALA) to form a complex (ALA-ginsenoside), its structure becomes more compact, and the hydrophobic interactions within the structure are stronger. Compared with ginsenoside alone, the complex exhibits stronger cytotoxicity and can enhance the bioaccessibility of ginsenoside [129]. Compared to primary saponins, secondary saponins are formed through structural changes such as hydrolysis, dehydration, or microbial conversion, can have a higher bioavailability [130, 131]. Following deglycosylation by artificial gastric acid, ginsenoside Rh2 exhibits superior intestinal absorption compared to its precursor ginsenosides, Rb1 and Rb2 (which have bioavailability of 0.78% and 0.08%, respectively). The bioavailability of ginsenoside Rh2 is significantly higher, reaching approximately 5% in rats and 15–25% in dogs [132].

The use of novel formulation technologies such as drug nanoparticles, drug emulsion droplets, and targeted formulations can also significantly improve the bioavailability of saponins. Compared with the amorphous saponin, the microcrystals prepared from akebia saponin D have increased the Peak Concentration (C_max_) from 75.5 ± 21.7 ng/mL to 409.9 ± 88.7 ng/mL, and the Area Under Curve (AUC) for 0–20 h also has increased from 528.4 ± 87.4 ng·h/mL to 2299.8 ± 217.4 ng·h/mL, representing a 4.3-fold improvement [133]. By incorporating ginsenosides Rh1 and Rh2 into self-microemulsifying systems (SME-1 and SME-2) containing intestinal cytochrome P450, P-glycoprotein inhibitory excipients, and only P-glycoprotein inhibitory excipients, the uptake of Rh1 and Rh2 by Caco-2 cells is increased by 1.69-fold and 1.62-fold, and 1.79-fold and 1.82-fold respectively compared with the free components. In in vivo experiments, after administration of the two emulsions prepared with the two saponins respectively, the areas under the drug concentration curves are significantly increased [134]. Targeted formulations can improve the bioavailability of drugs by selectively releasing the drugs at specific sites. When total saponins of Pulsatilla genus are prepared into a pH-dependent colon-targeted powder, this formulation reduces the release of the saponins in the stomach and small intestine, thereby increasing the drug concentration in the colon. It prolongs the peak time and residence time of the formulation in vivo, lowers the Cmax, and exhibits a certain lag effect, thus improving bioavailability [135]. The inclusion complex prepared jointly from ginsenoside IH901, β-cyclodextrin and hydroxypropyl β-cyclodextrin mainly releases the drug in the intestinal fluid. After rats are orally administered with the inclusion complex, its C_max_ is 2.8 times higher than that of pure IH901. According to the calculation of absolute bioavailability based on AUC_oral_/AUC_i.v_., the bioavailabilities of pure IH901 and the inclusion complex are 3.52% and 6.57% respectively [136]. The intestinal-targeted oral nanoparticles of jujuboside B (JuB) modified with L-carnitine were prepared using polyethylene glycol and poly(lactic-co-glycolic acid) (PEG-PLGA). In vivo pharmacokinetic experiments in rats showed that, compared with JuB, the relative bioavailability of the L-carnitine-modified JuB-PEG-PLGA particles increased to 159.04%, and they could enhance the uptake by Caco-2 cells [137].

### Haemolytic toxicity

Due to the special structure of saponins, the hydrophobic aglycone parts of some saponin substances can bind to cholesterol on the erythrocyte membrane to form complexes. At low concentrations, it may change the permeability of the membrane, and at high concentrations, it will damage the integrity of the membrane, leading to hemolysis [138]. The existence of this characteristic has greatly restricted the application and development of saponin-based injectable drugs. However, since injectable drugs have irreplaceable advantages such as rapid onset of action and accurate dosage, it is necessary to effectively reduce the risks through certain reasonable designs or adjustments of the administration methods.

Tests for the haemolytic activity of the synthesized semisynthetic saponins—diosgenyl β-D-glycosaminosides (D-gluco and D-galacto) hydrochloride salts and their N-alkyl analogues—show that these saponins exhibit no haemolytic toxicity towards human erythrocytes; researchers observed no haemolysis even when they exposed erythrocytes to saponins at a concentration of 256 µg/mL [139]. When the concentration of echinoside A in sea cucumbers is greater than 15 mg/L, the hemolysis rate reaches 100%. When the concentration of ds-echinoside A (the desulfurized product of echinoside A) is 15 mg/L, the hemolysis rate is reduced to approximately 50%. This indicates that the desulfurization reaction can reduce the hemolytic activity of echinoside A [140]. Dioscin has a very strong hemolytic toxicity. Even at a concentration of only 2.5 µg/mL, dioscin can make the hemolysis rate reach 18.5%. After it is combined with cholesterol to prepare a nanocomplex, the hemolysis degree of 2.5 µg/mL dioscin-cholesterol is much lower than 5% [141]. After escin Ia (EIa) is prepared into EIa- cholesterol, the total haemolytic complement activity (CH50) increases from 20.08 µg/mL to 910.60 µg/mL, which significantly reduces the hemolytic toxicity [141]. Paris saponin VII has a good anti-tumor effect on colorectal cancer. However, due to its lack of selective distribution in the body, it will lead to severe hemolytic reactions. When it is prepared into colorectal cancer-targeted calcium phosphate nanoparticles, it is found that it has almost no obvious effect on the morphology of red blood cells, and there is no obvious generation of red blood cell fragments, thus reducing the hemolytic toxicity of paris saponin VII [142]. To avoid the hemolytic toxicity of polyphyllin D, encapsulation with solid lipid nanoparticles (SLN) can achieve lower toxic and side effects through delivery to specific sites. In in vitro hemolysis experiments, polyphyllin D-loaded SLN showed a remarkable effect of reducing hemolysis [143].

**Fig. 7:**
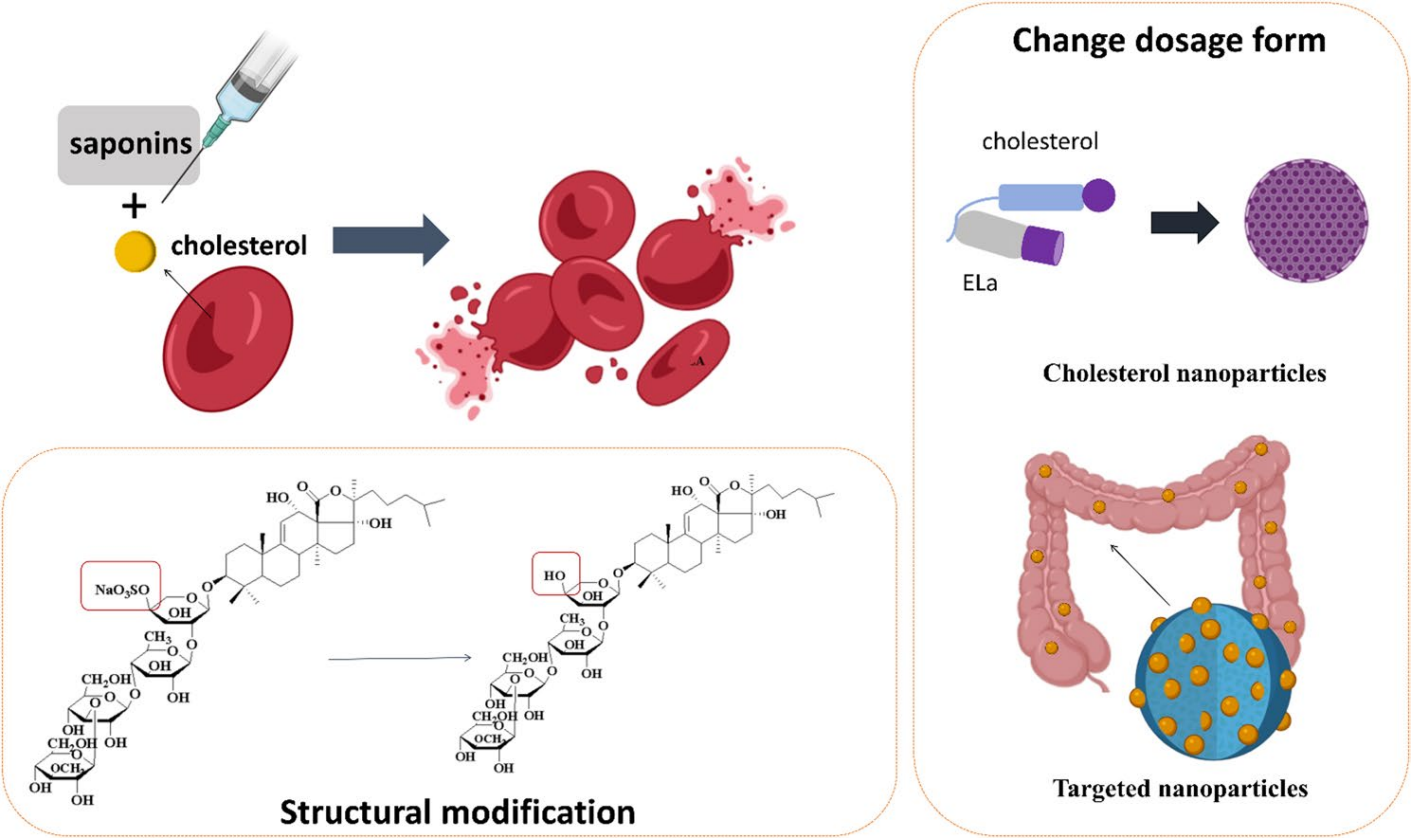
Strategies for reducing hemolytic toxicity

The hemolytic property of saponin-based drugs originates from their interaction with the cell membrane. However, through structural and formulation innovations and adjustments, the risks can be effectively avoided (Fig. 7). In the process of drug development, it is necessary to combine safety evaluation with the requirements of therapeutic efficacy and balance the therapeutic value and potential toxicity of these drugs.

### Discussion

This section focuses on the core limitations of saponins in antitumor therapy and their solutions, emphasizing the potential of key strategies (structural modification, formulation redesign). Low bioavailability and hemolytic toxicity represent the two primary bottlenecks restricting the clinical translation of saponins. The aforementioned strategies demonstrate significant advantages and application prospects based on their mechanisms of action and experimental outcomes.

Among these key strategies, nanomedicine technology demonstrates the most promising outlook, as it simultaneously addresses core limitations while offering broad applicability. Taking akebia saponin D as an example, microcrystalline nanoparticles increased its Cmax by 5.4-fold and AUC by 4.3-fold, significantly improving bioavailability. For polyphyllin D, solid lipid nanoparticles (SLNs) effectively reduced hemolytic toxicity through targeted delivery. L-carnitine-modified PEG-PLGA nanoparticles elevated jujuboside B’s bioavailability to 159.04%. Their advantages include flexible targeting (e.g., colonic, tumor-targeting) and the ability to shield saponins from gastrointestinal degradation while reducing non-specific interactions with red blood cell membranes.

Esterification holds significant potential as a structural modification strategy for enhancing bioavailability. Modification of ginsenoside Rh2 into Rh2 octyl ester increased intestinal permeability by 9.3-fold and enhanced cellular uptake; maleimidocaproic acid-modified Rh2 improved solubility and antitumor activity. This demonstrates that introducing lipophilic groups effectively overcomes the membrane permeability limitations restricting oral absorption of most hydrophilic saponins. However, this strategy primarily enhances absorption by optimizing physicochemical properties, lacking direct evidence of reduced hemolytic effects.

Notably, combined approaches (e.g., structural modification + formulation optimization) can further enhance efficacy: for instance, reducing diosgenin’s hemolytic toxicity through cholesterol nanocomplex formation achieves integration of structural interactions and nanodelivery. Future research should prioritize optimizing nanodelivery systems for injectable formulations while exploring combined strategies to balance bioavailability, hemolytic safety, and antitumor efficacy, thereby accelerating the clinical translation of saponin-based drugs.

## Conclusions

This review comprehensively elaborates the intrinsic antitumor effects and synergistic value of natural saponins in combination chemotherapy, as well as the bottlenecks and solutions for their clinical translation. Natural saponins isolated from medicinal plants exert potent anti-tumor effects through multiple pathways, and their combination with chemotherapeutic agents is particularly promising—this strategy not only enhances the cytotoxicity of chemotherapeutic drugs against tumor cells but also mitigates chemotherapy-induced toxicity, reverses tumor drug resistance, and strengthens host anti-tumor immunity. These synergistic advantages closely relate to the complementary mechanisms between saponins and chemotherapeutic agents: for instance, saikosaponin A can inhibit docetaxel-induced QCC proliferation; ginsenoside Rh4 can suppress the expression of efflux pumps upregulated by adriamycin. Therefore, the most promising saponin combination therapies to date appear to be those that achieve mechanistic complementarity by targeting the weaknesses of chemotherapeutic agents.

Despite the proven potential of saponins, their clinical translation in cancer treatment is still constrained by two key challenges: limited bioavailability caused by poor solubility and absorption, and the lack of systematic research on their combined application with chemotherapeutic agents. Currently, only a handful of saponin-based drugs (e.g., ginsenoside Rg3 preparations) have been approved for clinical anti-tumor applications, highlighting the significant gap between preclinical research prospects and clinical practical applications. To break through these bottlenecks, structural modification and advanced formulation design have emerged as effective solutions: structural modification can optimize the pharmacokinetic properties of saponins, while formulation strategies—especially nanotechnology-based drug delivery systems (e.g., poly(lactic-co-glycolic acid) nanoparticles, liposomes)—can significantly improve their solubility, bioavailability, and tumor targeting, thereby reducing off-target toxicity and enhancing therapeutic efficacy. Recent studies emphasize that the future development of saponin-based anti-tumor therapy is inextricably linked to the advancement of these delivery technologies, which will unlock the full therapeutic potential of saponins.

Importantly, although combining saponins with chemotherapeutic agents has demonstrated potent anti-tumor activity and tumor specificity (with minimal damage to normal cells), researchers have not yet fully elucidated the underlying mechanisms of their specific effects. In addition, current research on saponin-chemotherapeutic combination therapies remains fragmented, with inconsistent research focuses across different saponins and chemotherapeutic agents, and a lack of systematic exploration into their synergistic mechanisms. To address these critical knowledge gaps and accelerate the translational application of saponin-based combination therapies in clinical oncology, future research should prioritize three specific, high-impact directions with clear translational potential. Firstly, conduct high-throughput screening and preclinical validation of saponin-chemotherapeutic drug pairs across different tumor types, to identify optimal combinations that achieve mechanistic complementarity by targeting the inherent limitations of chemotherapeutic agents (e.g., drug resistance, off-target toxicity) and establish a standardized database for rational regimen design; Secondly, apply integrated multi-omics approaches (transcriptomics, proteomics, metabolomics) and single-cell analysis to elucidate the molecular basis of saponins’ tumor-selective cytotoxicity and the synergistic interaction mechanisms between saponins and chemotherapeutics, clarifying key signaling networks and cellular targets that drive their anti-tumor effects while distinguishing their differential actions on tumor cells and normal tissues; Thirdly, advance the development of novel saponin formulations (e.g., nanoparticle-encapsulated, receptor-targeted liposomes, pH-responsive prodrugs) and initiate phase I/II clinical trials combining these optimized formulations with standard chemotherapeutic regimens, focusing on evaluating their in vivo bioavailability, safety profiles (especially hemolytic toxicity and organ tolerance), and clinical efficacy in cancer patients, while establishing unified evaluation criteria for the therapeutic potential of saponin-based combination therapies.

In summary, natural saponins play an irreplaceable role in anti-tumor therapy, and their combined application with chemotherapeutic agents has opened up new pathways for cancer treatment by overcoming the limitations of conventional chemotherapy (e.g., drug resistance, immunosuppression, high toxicity). With the in-depth development of research on structural modification, formulation optimization, and mechanism exploration, the integration of natural saponins with Western chemotherapeutic agents will become increasingly refined. This integration not only is expected to significantly improve the efficacy and safety of anti-tumor treatments but also can provide more personalized treatment options for cancer patients. Furthermore, the growing demand for effective and safe anti-tumor therapies will drive the innovative development of the natural drug industry, promote the research and development as well as clinical translation of saponin-based drugs, and jointly shape a new pattern of comprehensive cancer treatment in the future.

## Supplementary Information

Supplementary Material 1.

## Data Availability

No datasets were generated or analysed during the current study.

## Abbreviations

AKI: acute kidney injury
ALA: alpha-lactalbumin
AML: Acute myeloid leukemia
AP-BL: Apical-Basolateral
AUC: Area Under Curve
CH50: total haemolytic complement activity
C_max_: Peak Concentration
CPT-11: irinotecan
CTX: Cyclophosphamide
EIa: escin Ia
EMT: epithelial-mesenchymal transition
GDP: Gross Domestic Product
IARC: international agency for research on cancer
IC_50_: half maximal inhibitory concentration
ICD: immunogenic cell death
JuB: jujuboside B
LDH: lactate dehydrogenase
MMP: mitochondrial membrane potential
NSCLC: non-small cell lung cancer
PAPP: apparent permeability coefficient
PEG-PLGA: polyethylene glycol and poly(lactic-co-glycolic acid)
PPI: polyphyllin I
PSA: pulsatilla saponin A
QCC: quiescent cancer cells
Rh2-O: derivative Rh2 octyl ester
ROS: reactive oxygen species
SLN: solid lipid nanoparticles
Teffs: effector T cells
TME: tumor microenvironment
