# Supplementary material for: Research status and challenges of anti-tumor effects of natural saponins and their combination therapy
Source: J Egypt Natl Canc Inst. 2026 May 18;38:16. doi: 10.1186/s43046-026-00354-x (PMC13313304; doi:10.1186/s43046-026-00354-x)
Supplement: Supplementary file 1 — Supplementary Material 1. [file 43046_2026_354_MOESM1_ESM.pdf]

**Table S1 Anti-tumor effects of triterpenoid saponins in natural products.**

| Family name | Source | Component | Cancer species | Effect | References |
|---|---|---|---|---|---|
| Araliaceae | *Panax ginseng* | Ginsenoside CK | Gastric cancer | Cytotoxic, inducing cancer cell apoptosis.<br>Bax、Caspase-3 ↑<br>CyclinB1、CyclinD1、Bcl-2、p-PI3K、p-AKT、NF-κB p65、p-IκBα ↓ | [31] |
| | | | Liver cancer | Cytotoxicity, inhibition of cancer cell proliferation, G0/G1 phase arrest, suppression of ATP production, inhibition of cellular glycolysis, and suppression of tumor growth in experimental animals.<br>PHD、RACK1 ↑<br>Bclaf1、HIF-1α、HSP70、HSP90、pVHL、GLUT1、HK2、LDHA、PDK1 ↓ | [32] |
| | | | | Cytotoxicity, induction of cancer cell apoptosis, inhibition of glycolysis.<br>pro-Caspase 3、pro-PARP、p-AKT、p-mTOR、c-Myc、HK2、PKM2 ↓ | [33] |
| | | | | Cytotoxicity, inhibition of cancer cell proliferation, increased thermal stability of Annexin A2, reduced interaction between Annexin A2 and p50.<br>Caspase 3、Caspase 9 ↑<br>IL-6、X-IAP， c-IAP1， c-IAP2、Survivin ↓ | [34] |
| | | | Lung cancer | Cytotoxicity, induction of cancer cell apoptosis, inhibition of tyrosine kinase activity, and suppression of tumor growth in experimental animals.<br>Caspase-9 ↑<br>EGFR、p-EGFR、p-B-RAF、p-C-RAF、p-ERK、p-MEK、p-STAT3、BcL-Xl、Survivin ↓ | [35] |

| | | | | | |
|---|---|---|---|---|---|
| | | Ginsenoside Rg3 | Colon cancer | Cytotoxicity, inhibition of cancer cell migration and invasion, suppression of EMT, and inhibition of tumor growth and metastasis in experimental animals.<br>E-cadherin ↑<br>Vimentin、Snail、NICD、Hes1、Notch1、Notch2、Notch3 ↓ | [36] |
| | | | | Cytotoxicity, induce mitochondrial damage and autophagy in cancer cells, enhance the interaction between PINK1 and Parkin proteins.<br>LC3-II、PINK1、Parkin、GAPDH、p62、ubiquitinated GAPDH ↑<br>TIM23 、VDAC1、MFN2、ULK1 ↓ | [37] |
| | | | Melanoma | Cytotoxicity, inhibition of cancer cell proliferation, inhibition of tumor growth in experimental animals.<br>FUT4、Lewis Y、UEA、p-EGFR、p-ERK1/2 ↓ | [38] |
| | | | NSCLC | Cytotoxicity, minor DNA damage.<br>VRK1、P53BP1 ↑ | [39] |
| Campanulaceae | *Platycodon grandiflorus* | Deapioplatycodin D | Liver cancer | Cytotoxicity, G2/M phase arrest, induces autophagy in cancer cells, leading to a decrease in mitochondrial membrane potential.<br>LC3-II、P62、BNIP3L、P21、ROS、γ-H2A.X、IL-6、IL-8、TGFβ、MMP-3、CXCL-1、IGFBP3 ↑<br>CyclinA2、Lamp1、cathepsin D、SNAP29-Syntaxin17-VAMP8、CDK 1、p-RB、E2F1 ↓ | [144] |
| | | Platycodin D | Acute myeloid leukemia | Cytotoxicity, induction of cancer cell apoptosis, reduction of mitochondrial membrane potential, G0/G1 cycle arrest.<br>Cleaved-PARP、Cleaved-Caspase-3、cytosolic cytochrome C、BAK、BIM ↑<br>p-Akt、p-GSK3β、p-S6、p-ERK ↓ | [40] |

| | | | | | |
|---|---|---|---|---|---|
| | | | Adrenal pheochromocytoma | Cytotoxicity, induction of cancer cell apoptosis, induction of DNA fragmentation, induction of mitochondrial membrane potential reduction, G0/G1 cycle arrest, induction of autophagy.<br>ROS、Caspase 3、Bid ↑<br>pro-Caspase 7、Bcl-2、Bcl-xL ↓ | [41] |
| | | | Liver cancer | Cytotoxicity, inducing escape of endocytosed EGFP-HBP from endosomes while damaging vesicle membranes, promoting redistribution of free cholesterol to enhance MHBP's antitumor activity, and facilitating cancer cell apoptosis.<br>MHBP、p-JNK、p-p38、Cleaved-Caspase-3、Cleaved-Caspase-8、Cleaved-Caspase-9 ↑<br>cathepsin B、cathepsin D、p-Akt、p-ERK1/2 ↓ | [42] |
| | | | | Cytotoxicity, inducing apoptosis and autophagy in cancer cells, and inhibiting tumor growth in experimental animals.<br>Bax/ Bcl-2、Cleaved-PARP、Caspase-3、LC3-II、p-ERK1/2、p-c-Jun ↑<br>Bcl-2 ↓ | [43] |
| Caryophyllaceae | *Saponaria officinalis* | Saponaria officinalis saponin 1 | Lung cancer | Cytotoxicity, induction of cancer cell apoptosis, G2/M phase arrest, induction of mitochondrial membrane potential depolarization, induction of mitochondrial autophagy.<br>Caspase-3、Caspase-8、Caspase-9、Cleaved-PARP、ROS ↑<br>Bcl-2 ↓ | [145] |
| | | SO1406 | triple-negative breast cancer | | |
| | *Vaccaria segetalis* | Segetoside I | Liver cancer | Induction of cancer cell apoptosis, dose-dependent DNA fragmentation, inhibition of cancer cell migration.<br>Bax ↑ | [146] |

| | | | | | |
|---|---|---|---|---|---|
| | | | | Bcl-2 ↓ | |
| Chenopodiaceae | *Salicornia bigelovii* | Bigelovii C | Breast cancer | Cytotoxicity, induction of cancer cell apoptosis, induction of decreased mitochondrial membrane potential.<br>Bax、Cleaved-Caspase-7、Cleaved-Caspase-9、Cleaved-PARP ↑<br>Bcl-2 ↓ | [147] |
| Cucurbitaceae | *Bolbostemma paniculatum* | Tubeimoside-1 | Prostatic cancer | Cytotoxicity, induction of cancer cell apoptosis, G0/G1 phase arrest, reduction of mitochondrial membrane potential.<br>ROS、p- ASK-1、p-JNK、 p-p38、CHOP、Bax、Cleaved-Caspase-3、p53、p21 ↑<br>Thioredoxin、Bcl-2、cyclin E、cdk2 ↓ | [44] |
| | | | Lung cancer | Cytotoxicity, inhibition of cancer cell proliferation, induction of mitochondrial fission, induction of autophagy, inhibition of lysosomal acidification, blocking of autophagic flux, induction of reduced mitochondrial membrane potential, suppression of tumor growth in experimental animals.<br>ROS、LC3-II、p62、cathepsin B、Bax、cytoplasmic cytochrome C、Cleaved-PARP、Cleaved-Caspase-3 ↑<br>p-DRP1、mature cathepsin D、V-ATPase activity ↓ | [45] |
| | | | Cervical carcinoma | Cytotoxicity, inhibition of cancer cell proliferation, induction of apoptosis, induction of autophagy, suppression of autophagic flux, inhibition of lysosomal activity, suppression of tumor growth in experimental animals.<br>Cleaved-Caspase-3、Cleaved-PARP、LC3-II、Beclin 1、Atg5、p-AMPK、p-ACC、p62、LAMP1、LAMP2、 RAB5、RAB7 ↑ | [46] |
| | | | Breast cancer | Activate autophagy and induce apoptosis in cancer cells. | [47] |

| | | | | | |
|---|---|---|---|---|---|
| | | | | LC3-II、p-eEF2、Cleaved-PARP、Cleaved-Caspase-3 ↑<br>p-Akt(Ser473、Thr308)、p-p70S6K、Mcl-1、Bcl-xL、Bcl-2 ↓ | |
| | *Gynostemma pentaphyllum* | Gypenoside L | Liver cancer | Cytotoxicity, inducing non-apoptotic cytoplasmic vacuolation death, inducing autophagy, blocking autophagic flux, inducing endoplasmic reticulum stress.<br>LC3-II、p62、PDI、IRE1-α、Ero1-Lα、PERK、$Ca^{+}$ ↑ | [148] |
| | | | Liver cancer, Esophageal cancer | Induce cancer cell senescence, inhibit cancer cell proliferation, S phase arrest.<br>IL-1α、IL-6、TIMP-1、CXCL-1、CXCL-2、p21、p18、p27、p-CHK2、p-AKT、p-p38、p-ERK、p-NF-κB ↑<br>CDK2、CDK4、CDK6、cyclin D1 ↓ | [149] |
| | | Gypenoside LI | Breast cancer | Cytotoxicity, inhibition of cancer cell proliferation, inhibition of cancer cell migration and invasion, induction of apoptosis, G0/G1 phase arrest.<br>Bax、cytochrome C ↑<br>MMP-2、MMP-9、PARP-1、Bcl-2、CDK2、CDK4、cyclin D1、ERCC6L、E2F1 ↓ | [150] |
| | | | Lung cancer | Cytotoxicity, induction of cancer cell apoptosis, reduction of mitochondrial membrane potential, G2/M phase arrest, inhibition of cancer cell migration and invasion.<br>ROS ↑<br>pro-Caspase 8、MMP-2、MMP-9、CDK1 ↓ | [151] |
| Leguminosae | *Astragalus membrana* | Astragaloside IV | Breast cancer | Cytotoxicity, inhibition of cancer cell proliferation, inhibition of cancer cell migration, inhibition of tumor growth in experimental animals | [152] |

| | | | | | |
|---|---|---|---|---|---|
| | *ceus* | | | Vav3、GTP-Rac1、p-ERK1/2、p-JNK、MMP-2、MMP-9↓ | |
| | | | Cervical carcinoma | Cytotoxicity, inhibition of cancer cell invasion, induction of autophagy, suppression of tumor growth in experimental animals.<br>LC3-II、DCP1A、TMSB4X、Atg7、Atg12↑<br>MGST3、AKR1C2、ERL1N1↓ | [153] |
| | | | Colorectal cancer | Cytotoxicity, inhibition of cancer cell proliferation, G0/G1 phase arrest.<br>miR-29c↑<br>cyclin D1、CDK4、B7-H3、p-p65↓ | [154] |
| | Gleditsiae fructus abnormalis | Gleditsia Saponin C | Lung cancer | Inhibits cancer cell proliferation, induces apoptosis, induces cytochrome C release.<br>Cleaved-Caspase-8 、 Cleaved-Caspase-9 、 Cleaved-Caspase-3 、 Cleaved-Caspase-7；Cleaved-PARP；Bad；Bax；IκBα↑<br>Bcl -xl、Bcl-2、p-PI3K、p- Akt、p-p38、p-ERK、p- JNK、p- IκBα↓ | [155] |
| Marine animals | Sea cucumber | Holothurin A | Prostatic cancer | Cytotoxicity, inhibition of cancer cell migration and invasion.<br>E-cadherin↑<br>RUNX1、vimentin、MMP-2、MMP-9、N-cadherin、vimentin、twist1、slug、snail、p-Akt、p-ERK、p-JNK、p-P38↓ | [156] |
| Meliaceae | *Dysoxylum cumingianum* | Cumingianoside A | Melanoma | Inhibit cancer cell growth and proliferation, induce apoptosis, G2/M phase arrest, induce autophagy.<br>Cleaved-Caspase-3、Cleaved-Caspase-7、Cleaved-PARP、IRE1α、ATG13、BECN1、ATG5、ATG12、LC3B、LAMP2、LC3↑<br>cyclin B1、CDK1、p-CDK1(Thr161)、CDC25C 、p-Rb(Ser808/811)、E2F1、pro-Caspase3↓ | [157] |

| | | | | | |
|---|---|---|---|---|---|
| Pedunculaceae | *Phytolacca acinosa* | Esculentoside A | Breast cancer | Inhibits cancer cell growth, induces apoptosis, and suppresses tumor growth in experimental animals.<br>Bax、Cleaved-Caspase-3 ↑<br>ALDH1A1、Sox2、Oct4、Bcl-2、IL-6、p-STAT3 (Tyr705)、p-STAT3 (Ser727) ↓ | [158] |
| Primulaceae | *Androsace umbellata* | Afrocyclamin A | Prostatic cancer | Inhibits cancer cell growth (with no significant effect on normal cells), G0/G1 phase arrest, induces apoptosis, promotes autophagosome formation, suppresses cancer cell migration and invasion, and inhibits tumor growth in experimental animals.<br>p53、p21Waf1/Cip1、p27kip1、Bax、Cleaved-PARP、Cleaved-Caspase3、cytochrome C、Bax/Bcl-2、LC3I、LC3II、Beclin-1、Atg5、Atg7 ↑<br>cyclin E、cyclin D、cyclin B、CDK2、CDK4、MDM2、Bcl-2、MMP-2、MMP-9、p-PI3k、p-Akt、p-mTOR、Ki67(in mouse tumors) ↓ | [159] |
| | | Saxifragifolin C | Breast cancer | Inhibit cancer cell growth and induce apoptosis.<br>ROS、Bax、cytoplasmic cytochrome C、cytoplasmic AIF、Cleaved-Caspase-8、Cleaved-Caspase-3、p-ERK 1/2、p-p38、p-JNK ↑<br>Bcl-2、Bcl-xL、mitochondrial cytochrome C、mitochondrial AIF ↓ | [160] |
| Quillajaceae | *Quillaja saponaria Molina* | QS-21 | Gastric cancer | Inhibits cancer cell growth (with no significant effect on normal cells), disrupts cancer cell membrane integrity, induces apoptosis and DNA fragmentation, and binds to the pro-apoptotic protein Bid.<br>Caspase 3 and 7 activity ↑ | [161] |
| Ranunculaceae | *Anemone raddeana* | Raddeanin A | Breast cancer | Enhance autophagy flux, suppress cancer cell viability, and induce apoptosis.<br>LC3-II、p-eEF2、Cleaved-Caspase-3、Cleaved-PARP ↑ | [48] |

| | | | |
|---|---|---|---|
| | | p-S6K、p-Akt、Bcl-xL、Mcl-1、Bcl-2 ↓ | |
| | Colorectal cancer | Inhibits cancer cell proliferation and migration, suppresses angiogenesis, and exhibits structural docking with VEGFR2 kinase.<br>p-VEGFR2、p-plc - γ - 1、p-JAK2、p-FAK、p-Src、p-Akt ↓ | [49] |
| | | Cytotoxicity, induction of cancer cell apoptosis, reduction of mitochondrial membrane potential, inhibition of cancer cell proliferation, G0/G1 phase arrest, and suppression of tumor growth in experimental animals.<br>Bax、Cleaved-Caspase-3、Cleaved-PARP、p-β-catenin ↑<br>Bcl-2、c-Myc mRNA、Cyclin D1 mRNA、β-catenin、p-GSK-3β、p-Akt、p- LRP6、p-IKBα ↓ | [50] |
| | Osteosarcoma | Cytotoxicity, inhibition of cancer cell proliferation, induction of apoptosis, suppression of tumor growth in experimental animals.<br>ROS、Cleaved PARP、Cleaved-Caspase- 8、Cleaved-Caspase- 9、Cleaved-Caspase- 3、Bax、p-ERK1/2、p-JNK、p-c- Jun ↑<br>Bcl- 2、Bcl- xl、p-STAT3 ↓ | [51] |
| | | Cytotoxicity, inhibition of cancer cell proliferation, induction of apoptosis.<br>Cleaved-PARP、Bax ↑<br>Bcl-2、Bcl-xL、p-STAT3、p-JAK2、MDR1 ↓ | [52] |
| | Prostatic cancer | Depends on the androgen receptor (AR) to inhibit cancer cell growth and suppress AR signaling.<br>AR-FL、AR-V and their mRNA ↓ | [53] |

| | | | | |
|---|---|---|---|---|
| *Pulsatilla chinensis* | α-Hederin | Colon cancer | Cytotoxicity, inhibition of cancer cell proliferation, induction of apoptosis, induction of endoplasmic reticulum stress, induction of autophagy, blockade of autophagy flux.<br>Cleaved-PARP、Cleaved-Caspase-3、GRP78、PERK、eIF2α、p-eIF2α、IRE1、ATF6、calnexin、ATF4、Bax、p-IRE1、p-JNK、p-p38、LC3-II、p62 ↑<br>Bcl-2 ↓ | [54] |
| | | Gastric cancer | Inhibits cancer cell proliferation, G1 phase arrest, induces apoptosis, reduces mitochondrial membrane potential, and suppresses tumor growth in experimental animals.<br>P16、P21、P53、cdk2、Bax、Cleaved-Caspase-3、Cleaved-Caspase-8、Cleaved-Caspase-9、ROS ↑<br>Cyclin D1、Bcl-2、glutathione、ATP ↓ | [55] |
| | | Ovarian cancer | Inhibits cancer cell growth, induces nuclear fragmentation and chromatin condensation, triggers apoptosis, reduces mitochondrial membrane potential, G0/G1 phase arrest.<br>Caspase-9 activity、Caspase-3/7 activity ↑<br>Bcl-2↓ | [56] |
| | Pulsatilla saponin A | Liver cancer, pancreatic cancer | Inhibits the growth of human hepatocellular carcinoma and pancreatic cancer cells both in vitro and in vivo, induces DNA damage, G2 phase arrest, and apoptosis.<br>p53、cyclin B ↑<br>Bcl-2 ↓ | [162] |
| | Pulsatilla Saponin D | Glioblastoma multiforme | Inhibit cancer cell proliferation, suppress autophagy flux, reduce mitochondrial membrane potential, and increase lysosomal membrane | [163] |

| | | | | | |
|---|---|---|---|---|---|
| | | | | permeability.<br>IC3-II、p62、ROS ↑<br>IC3-I ↓ | |
| | | | — | Inhibit cancer cell growth, arrest the cell cycle, and induce apoptosis.<br>Cleaved PARP ↑<br>PARP、pro-Caspase-3、pro-Caspase -9、pro-Caspase -8 ↓ | [164] |
| Sapindaceae | *Aesculus chinensis* | Escin | Bladder cancer | Cytotoxicity, induction of cancer cell apoptosis, G2/M or G0/G1 phase arrest, reduction of mitochondrial membrane potential, inhibition of tumor growth in experimental animals.<br>Cleaved-Caspase-8 、 Cleaved-Caspase-9 、 Cleaved-Caspase-3 、 Cleaved-PARP、Fas、cytoplasmic cytochrome C、Cleaved-Bax、ROS、STAT3(low concentration) ↑<br>FADD、Bcl-2、BCL-xL、NF-κB/p65、STAT3(high concentration) ↓ | [165] |
| | | | Colorectal cancer | Cytotoxicity, inhibition of cancer cell proliferation, induction of apoptosis, induction of autophagy, suppression of tumor growth in experimental animals.<br>p-ATM、p-53BP1、γH2AX、ROS、p62、Cleaved-PARP、Cleaved-Caspase-9、LC3-II ↑ | [166] |
| | *Aesculus hippocastanum* | Aescin | Liver cancer, Colorectal cancer | Induce cancer cell apoptosis, activate autophagy, regulate autophagy flux.<br>LC3-II、Cleaved-PARP、Cleaved-Caspase-3、ATG5-ATG12、ROS、p-ATM(Ser1981)、p-AMPK(Thr172)、p-ULK1(Ser317)、Cleaved-Caspase-9 ↑ | [167] |

* "↑" and "↓" in the table indicate up and down regulation, respectively.

**Table S2 Anti-tumor effects of steroidal saponins in natural products.**

| Family name | Source | Component | Cancer species | Effect | References |
|---|---|---|---|---|---|
| Amaryllidaceae | *Allium chinense* | A-24 | Gastric cancer | Induce cancer cell apoptosis, inhibit migration, induce autophagy.<br>Cleaved-PARP-1、Cleaved-Caspase-3、Bax、ROS、LC3-II ↑<br>Bcl-2、p-mTOR、MMP-2 ↓ | [168] |
| Asparagaceae | *Anemarrhena asphodeloides* | Timosaponin AIII | Breast cancer | Induce cancer cell senescence, inhibit cancer cell proliferation, migration, and invasion.<br>miR-141、miR-200c ↑<br>BMI1 and its mRNA、H2AUb、c-Myc、PRC1 activity ↓ | [57] |
| | | | Lung cancer | Cytotoxicity, induction of cancer cell apoptosis (at high concentrations), induction of mitochondrial membrane potential reduction, induction of autophagy (at low and high concentrations) with activation of autophagic flux, and inhibition of tumor growth in experimental animals.<br>Cleaved-Caspase-3 、 Cleaved-Caspase-8 、 Cleaved-Caspase-9 、 Cleaved-PARP、cyt C、AIF、EndoG、Bax、LC3-II、Beclin 1、p-AMPK ↑<br>p-Erk1/2 ↓ | [58] |
| | | | NSCLC | Inhibits the growth and proliferation of cancer cells both in vivo and in vitro, causing G2/M cycle arrest, suppressing cell migration and invasion, and reducing mitochondrial membrane potential. Forms a complex with HSP90, further targeting and triggering the ubiquitination and degradation of GPX4, thereby inducing ferroptosis in cancer cells.<br>E-cadherin、ROS、HMOX-1、HSP90 ↑<br>VIM、SNAIL-2、SNAIL-1、MMP-9、FTL、GPX4、SLC40A1、SLC7A11 ↓ | [59] |

| | | | | | |
|---|---|---|---|---|---|
| | | | Pancreatic cancer | Inhibit cancer cell growth, causing G2/M phase arrest, and induce apoptosis.<br>p-JNK(high dose)、p-ERK1/2(low dose)、p21↑<br>ERK1/2、STAT3、p-ERK1/2、p-STAT3、Src、p-Src、p-JNK(low dose)、p-ERK1/2(high dose)、Bcl-2、cyclin D1、MMP-9、VEGF-1↓ | [60] |
| Dioscoreaceae | *Dioscorea polystachya* | Trillin | Liver cancer | Capable of traversing cell membranes to induce mitochondrial damage, endoplasmic reticulum stress, apoptosis, and autophagy in cancer cells.<br>LC3II/I、Beclin 1、Cleaved-Caspase3、9、IRE-1α、Caspase-12、Cleaved-PAPR↑<br>Bcl-2、p62↓ | [169] |
| | *Dioscorea tokoro* | Dioscin | Cervical carcinoma | Cytotoxicity, DNA damage induction, apoptosis induction.<br>ROS、$Ca^+$、cytoplasmic cytochrome C、Bax、Bak、Bid、p53、Caspase-3、Caspase-9↑<br>Bcl-2、Bcl-xL↓ | [61] |
| | | | Laryngocarcinoma | Cytotoxicity, causing S phase arrest, inducing apoptosis, inducing DNA damage, inhibiting cancer cell migration and invasion.<br>ROS、p53、p-JNK、p-p38、cytoplasmic cytochrome C、Bax、Cleaved-Caspase-3、Cleaved-Caspase-9↑<br>MMP-2、MMP-9、CDK2、Cyclin A、p-ERK1/2、Bcl-2↓ | [62] |
| | | | Liver cancer | Cytotoxicity, inhibition of cancer cell proliferation and migration.<br>connexin 43、ZO-1、claudin-1、E-cadherin↑<br>MKK3、p-Erk、p-p38、p-JNK、N-cadherin、Vimentin、Snail↓ | [63] |
| | | | Lung cancer | Cytotoxicity, inhibition of cancer cell proliferation, suppression of cancer cell migration and invasion, inhibition of EMT, induction of apoptosis, and suppression of tumor growth in experimental animals. | [64] |

| | | | | | |
|---|---|---|---|---|---|
| | | | | E-cadherin、Bax、Cleaved-Caspase-3、Cleaved-PARP、p-p38、p-HSP27、ROS ↑<br>N-cadherin、Vimentin、Bcl-2 ↓ | |
| | | | Osteosarcoma | Cytotoxicity, inhibition of cancer cell proliferation, causing G2/M phase arrest, induction of apoptosis, induction of decreased mitochondrial membrane potential, induction of cancer cell pyroptosis, inhibition of tumor growth in experimental animals.<br>P53、P21、p- Cdc2、p-ATM、Cleaved-PARP、Cleaved-Caspase-3、Cleaved-Caspase-8、Cleaved-Caspase-9、cytochrome C、Bax、p-p38、p-JNK、LDH release、GSDME – N ↑<br>Bcl-2、Ki-67 ↓ | [65] |
| | | | Prostatic cancer | Cytotoxicity, induction of cancer cell apoptosis, inhibition of proliferation, inhibition of cancer cell migration, inhibition of tumor growth in experimental animals.<br>p-SHP1、Caspase-3、Bad ↑<br>p-Erk1 /2 (T202/T204)、p-p38 (T182)、p-Akt (T326) ↓ | [66] |
| Liliaceae | *Ophiopogon japonicus* | DT-13 | Breast cancer | No cytotoxicity, inhibits cancer cell migration.<br>PLOD2 and its mRNA、P-STAT3、P-AKT ↓<br>When co cultured with fat：GP130、OBR ↓ | [170] |
| | | | Colorectal cancer | Inhibits cancer cell growth and proliferation, downregulates glucose uptake levels, and suppresses tumor growth in experimental animals.<br>p-AMPK ↑<br>lactic acid、intracellular ATP、GLUT1 mRNA、GLUT1 、GLUT3、GLUT4、HK2 and its mRNA、PFKM and its mRNA、LDHA and its mRNA、p-mTOR、p-P70S6K、p-4EBP1 ↓ | [171] |

| | | | | |
|---|---|---|---|---|
| | | Gastric cancer, NSCLC | Inhibit cancer cell proliferation and induce autophagy.<br>LC3-II、Beclin-1、Atg-7、Atg-3、Cleaved-Caspase-3、Cleaved-Caspase-9 ↑<br>Caspase-3、Caspase-9、p-PI3K、p-Akt、p-mTOR ↓ | [172] |
| | | Prostatic cancer | Inhibits cancer cell proliferation, induces apoptosis, reduces mitochondrial membrane potential, and suppresses cell migration and invasion.<br>Bax、Bad、cytoplasmic cytochrome C、Cleaved-Caspase-9、-3、Cleaved-PARP、E-cadherin ↑<br>Bcl-2、mitochondrial cytochrome C、p- Integrin β1、MMP-2/-9 activity、vimentin、p-Akt、p-mTOR、p-p70S6K、p-PDK1 ↓ | [173] |
| Rhizoma Paridis | Polyphyllin II | Colorectal cancer | Inhibits cancer cell growth, induces apoptosis, causes G1 cycle arrest, suppresses mitochondrial fission, and inhibits p65 nuclear translocation.<br>cyclin D1、c-Myc、p-DRP1、p-ERK、p-IKKα、p-p65 ↓ | [67] |
| | | Liver cancer | Inhibits cancer cell activity, induces apoptosis, induces a decrease in mitochondrial membrane potential, enhances mitochondrial membrane permeability, and causes S-phase cycle arrest.<br>ROS、p53、p21、Bax、cytoplasmic cytochrome C、Fas、Cleaved-Caspase-3、Cleaved-Caspase-8、Cleaved-Caspase-9、Cleaved-PARP ↑<br>cyclin A、cyclin E、CDK 2、Bcl-2、mitochondrial cytochrome C ↓ | [68] |
| | | Lung cancer | Induce autophagy and apoptosis in cancer cells.<br>LC3-II、Beclin-1、cytochrome C、Caspase 9、Cleaved-Caspase3、Bax、p-JNK ↑<br>p62、Bcl-2、p-PI3K、p-AKT、p-mTOR、ROS ↓ | [69] |

| | | | | | |
|---|---|---|---|---|---|
| Melanthiaceae | *Paris polyphylla* | Polyphyllin D | Breast cancer | Cytotoxicity, induction of cancer cell apoptosis, induction of autophagy, enhancement of Beclin1-Vps34 interaction, inhibition of tumor growth in experimental animals.<br>Bax、Cleaved-Caspase-3、Cleaved-Caspase-8、LC3-II、Beclin1、p-JNK、p-Bcl-2 ↑<br>Bcl-2、p62 ↓ | [70] |
| | | | Leukemia | Cytotoxicity, induction of cancer cell apoptosis, induction of decreased mitochondrial membrane potential, induction of differentiation of human chronic myeloid leukemia cells into monocytes or mature macrophages.<br>Bax、Cleaved-Caspase-3、CD14 ↑<br>Bcl-2、p210-Bcr/Abl ↓ | [71] |
| | *Trillium tschonoskii* | Paris saponin VII | Colorectal cancer | Inhibit cancer cell growth and proliferation, induce apoptosis.<br>Caspase-3 activity、Cleaved-Caspase-3、Cleaved-Caspase -9、Bax、cytoplasmic cytochrome C ↑<br>Mitochondrial membrane potential、Bcl-2、p-STAT3 ↓ | [174] |
| | | | Lung cancer | Inhibit cancer cell growth, migration, and invasion.<br>TIMP-1、TIMP-2、E-cadherin ↑<br>MMP-2、-9 activity ↓ | [175] |
| Solanaceae | *Solanum nigrum* | S-20 | Leukemia | Cytotoxicity, inhibition of cancer cell proliferation, G2/M phase arrest, induction of apoptosis, reduction of mitochondrial membrane potential.<br>p-cdc2、Myt1、Cleaved-Caspase-3、ROS、Beclin-1、LC3-II、p-ERK ↑<br>BCRP、ERK、P-gp、cyclin B1、p-wee1、p-histone H3、PARP-1、pro-Caspase-3、pro-Caspase-8、pro-Caspase-9、p62、p-JNK、 p-p38 ↓ | [176] |

* "↑" and "↓" in the table indicate up and down regulation, respectively.

# References in the supplementary materials

[144] Li YQ, Xiao PP, Li YR, Sun Y, Zhao HF, Sun JL, et al. Deapioplatycodin D promotes cell senescence induced by P21 through the mediation of incomplete mitophagy via BNIP3L. Biomedicine & Pharmacotherapy. 2024;178:117215.

[145] Takahashi N, Iguchi T, Kuroda M, Mishima M, Mimaki Y. Novel Oleanane-Type Triterpene Glycosides from the Saponaria officinalis L. Seeds and Apoptosis-Inducing Activity via Mitochondria. International Journal of Molecular Sciences. 2022;23(4):2047.

[146] Firempong CK, Zhang HY, Wang Y, Chen JJ, Cao X, Deng WW, et al. Segetoside I, a plant-derived bisdesmosidic saponin, induces apoptosis in human hepatoma cells in vitro and inhibits tumor growth in vivo. Pharmacological research. 2016;110:101-10.

[147] Guan FQ, Wang QZ, Wang M, Shan Y, Chen Y, Yin M, et al. Isolation, Identification and Cytotoxicity of a New Noroleanane-Type Triterpene Saponin from Salicornia bigelovii Torr. Molecules. 2015;20(4):6419-31.

[148] Zheng K, Liao CH, Li Y, Fan XM, Fan L, Xu H, et al. Gypenoside L, Isolated from Gynostemma pentaphyllum, Induces Cytoplasmic Vacuolation Death in Hepatocellular Carcinoma Cells through Reactive-Oxygen-Species-Mediated Unfolded Protein Response. Journal of Agricultural and Food Chemistry. 2016;64(8):1702-11.

[149] Ma JX, Hu XP, Liao CH, Xiao HT, Zhu QC, Li Y, et al. Gypenoside L Inhibits Proliferation of Liver and Esophageal Cancer Cells by Inducing Senescence.

Molecules. 2019;24(6):1054.

[150] Zu ML, Duan Y, Xie JB, Qi YS, Xie P, Borjigidai A, et al. Gypenoside LI arrests the cell cycle of breast cancer in G0/G1 phase by down-regulating E2F1. Journal of Ethnopharmacology. 2021;273:114017.

[151] Xing SF, Liu LH, Zu ML, Ding XF, Cui WY, Chang T, et al. The inhibitory effect of gypenoside stereoisomers, gypenoside L and gypenoside LI, isolated from Gynostemma pentaphyllum on the growth of human lung cancer A549 cells. Journal of Ethnopharmacology. 2018;219:161-72.

[152] Jiang K, Lu Q, Li Q, Ji YJ, Chen WL, Xue XH. Astragaloside IV inhibits breast cancer cell invasion by suppressing Vav3 mediated Rac1/MAPK signaling. International Immunopharmacology. 2017;42:195-202.

[153] Xia CL, He ZH, Cai YT. Quantitative proteomics analysis of differentially expressed proteins induced by astragaloside IV in cervical cancer cell invasion. Cellular & Molecular Biology Letters. 2020;25(1):25.

[154] Wang SX, Mou JG, Cui LS, Wang XG, Zhang ZQ. Astragaloside IV inhibits cell proliferation of colorectal cancer cell lines through down-regulation of B7-H3. Biomedicine & Pharmacotherapy. 2018;102:1037-44.

[155] Cheng Y, He WD, He YM. Gleditsia Saponin C Induces A549 Cell Apoptosis via Caspase-Dependent Cascade and Suppresses Tumor Growth on Xenografts Tumor Animal Model. Frontiers in Pharmacology. 2018;8:988.

[156] Janta S, Pranweerapaiboon K, Vivithanaporn P, Plubrukarn A, Chairoungdua A, Prasertsuksri P, et al. Holothurin A Inhibits RUNX1-Enhanced EMT in Metastasis

Prostate Cancer via the Akt/JNK and P38 MAPK Signaling Pathway. Marine Drugs. 2023;21(6):345.

[157] Cvetanova B, Shen YC, Shyur LF. Cumingianoside A, a Phyto-Triterpenoid Saponin Inhibits Acquired BRAF Inhibitor Resistant Melanoma Growth via Programmed Cell Death. Frontiers in Pharmacology. 2019;10:30.

[158] Liu CL, Dong LH, Sun Z, Wang L, Wang QP, Li HY, et al. Esculentoside A suppresses breast cancer stem cell growth through stemness attenuation and apoptosis induction by blocking IL-6/STAT3 signaling pathway. Phytotherapy Research. 2018;32(11):2299-311.

[159] Sachan R, Kundu A, Jeon Y, Choi WS, Yoon K, Kim IS, et al. Afrocyclamin A, a triterpene saponin, induces apoptosis and autophagic cell death via the PI3K/Akt/mTOR pathway in human prostate cancer cells. Phytomedicine. 2018;51:139-50.

[160] Kim KH, Kim JY, Kwak JH, Kim BO, Pyo S. Different apoptotic effects of saxifragifolin C in human breast cancer cells. Archives of Pharmacal Research. 2016;39(4):577-89.

[161] Guzmán L, Villalón K, Marchant MJ, Tarnok ME, Cárdenas P, Aquea G, et al. In vitro evaluation and molecular docking of QS-21 and quillaic acid from Quillaja saponaria Molina as gastric cancer agents. Scientific reports. 2020;10(1):10534.

[162] Liu Q, Chen WC, Jiao Y, Hou JQ, Wu QY, Liu YL, et al. Pulsatilla saponin A, an active molecule from Pulsatilla chinensis, induces cancer cell death and inhibits tumor growth in mouse xenograft models. Journal of Surgical Research.

2014;188(2):387-95.

[163] Hong JM, Kim JH, Kim H, Lee WJ, Hwang YI. SB365, Pulsatilla Saponin D Induces Caspase-Independent Cell Death and Augments the Anticancer Effect of Temozolomide in Glioblastoma Multiforme Cells. Molecules. 2019;24(18):3230.

[164] Chen Z, Duan HQ, Tong XH, Hsu PL, Han L, Morris-Natschke SL, et al. Cytotoxicity, Hemolytic Toxicity, and Mechanism of Action of Pulsatilla Saponin D and Its Synthetic Derivatives. Journal of Natural Products. 2018;81(3):465-74.

[165] Cheng CL, Chao WT, Li YH, Ou YC, Wang SS, Chiu KY, et al. Escin induces apoptosis in human bladder cancer cells: An in vitro and in vivo study. European Journal of Pharmacology. 2018;840:79-88.

[166] Wang Z, Chen Q, Li B, Xie JM, Yang XD, Zhao K, et al. Escin-induced DNA damage promotes escin-induced apoptosis in human colorectal cancer cells via p62 regulation of the ATM/γH2AX pathway. Acta Pharmacologica Sinica. 2018;39(10):1645-60.

[167] Li B, Wu GL, Dai W, Wang G, Su HY, Shen XP, et al. Aescin-induced reactive oxygen species play a pro-survival role in human cancer cells via ATM/AMPK/ULK1-mediated autophagy. Acta Pharmacologica Sinica. 2018;39(12):1874-84.

[168] Xu JW, Wang YH, Wang Y, Wang Z, He XJ. A-24, a steroidal saponin from Allium chinense, induced apoptosis, autophagy and migration inhibition in p53 wild-type and p53-deficient gastric cancer cells. Chemico-Biological Interactions. 2021;348:109648.

[169] Meng X, Dong HH, Pan YW, Ma L, Liu CX, Man SL, et al. Diosgenyl Saponin Inducing Endoplasmic Reticulum Stress and Mitochondria-Mediated Apoptotic Pathways in Liver Cancer Cells. Journal of Agricultural and Food Chemistry. 2019;67(41):11428-35.

[170] He JY, Wei XH, Li SJ, Quan XP, Li RM, Du HZ, et al. DT-13 suppresses breast cancer metastasis by modulating PLOD2 in the adipocytes microenvironment. Phytomedicine. 2019;59:152778.

[171] Wei XH, Mao TT, Li SJ, He JY, Hou XY, Li HY, et al. DT-13 inhibited the proliferation of colorectal cancer via glycolytic metabolism and AMPK/mTOR signaling pathway. Phytomedicine. 2019;54:120-31.

[172] Li HY, Sun L, De Carvalho EL, Li XX, Lv XD, Khan GJ, et al. DT-13, a saponin monomer of dwarf lilyturf tuber, induces autophagy and potentiates anti-cancer effect of nutrient deprivation. European Journal of Pharmacology. 2016;781:164-72.

[173] Wang ZM, Wang YY, Zhu S, Liu Y, Peng X, Zhang SL, et al. DT-13 Inhibits Proliferation and Metastasis of Human Prostate Cancer Cells Through Blocking PI3K/Akt Pathway. Frontiers in Pharmacology. 2018;9:1450.

[174] Zhou HP, Sun Y, Zheng HN, Fan L, Mei QB, Tang Y, et al. Paris saponin VII extracted from trillium tschonoskii suppresses proliferation and induces apoptosis of human colorectal cancer cells. Journal of Ethnopharmacology. 2019;239:111903.

[175] Fan L, Li YH, Sun Y, Han J, Yue ZG, Meng J, et al. Paris Saponin VII Inhibits the Migration and Invasion in Human A549 Lung Cancer Cells. Phytotherapy

Research. 2015;29(9):1366-72.

[176] Wang Y, Xu JW, Wang YH, Xiang LM, He XJ. S-20, a steroidal saponin from the berries of black nightshade, exerts anti-multidrug resistance activity in K562/ADR cells through autophagic cell death and ERK activation. Food & Function. 2022;13(4):2200-15.
